# Modulation of human corticospinal excitability by paired associative stimulation

**DOI:** 10.3389/fnhum.2013.00823

**Published:** 2013-12-03

**Authors:** Richard G. Carson, Niamh C. Kennedy

**Affiliations:** ^1^Trinity College Institute of Neuroscience and School of Psychology, Trinity College DublinDublin, Ireland; ^2^School of Psychology, Queen's University BelfastBelfast, UK; ^3^School of Rehabilitation Sciences University of East AngliaNorwich, UK

**Keywords:** long-term potentiation, long-term depression, transcranial magnetic stimulation, peripheral nerve stimulation, human, cortex, spike-timing dependent plasticity, translational neuroscience

## Abstract

Paired Associative Stimulation (PAS) has come to prominence as a potential therapeutic intervention for the treatment of brain injury/disease, and as an experimental method with which to investigate Hebbian principles of neural plasticity in humans. Prototypically, a single electrical stimulus is directed to a peripheral nerve in advance of transcranial magnetic stimulation (TMS) delivered to the contralateral primary motor cortex (M1). Repeated pairing of the stimuli (i.e., association) over an extended period may increase or decrease the excitability of corticospinal projections from M1, in manner that depends on the interstimulus interval (ISI). It has been suggested that these effects represent a form of associative long-term potentiation (LTP) and depression (LTD) that bears resemblance to spike-timing dependent plasticity (STDP) as it has been elaborated in animal models. With a large body of empirical evidence having emerged since the cardinal features of PAS were first described, and in light of the variations from the original protocols that have been implemented, it is opportune to consider whether the phenomenology of PAS remains consistent with the characteristic features that were initially disclosed. This assessment necessarily has bearing upon interpretation of the effects of PAS in relation to the specific cellular pathways that are putatively engaged, including those that adhere to the rules of STDP. The balance of evidence suggests that the mechanisms that contribute to the LTP- and LTD-type responses to PAS differ depending on the precise nature of the induction protocol that is used. In addition to emphasizing the requirement for additional explanatory models, in the present analysis we highlight the key features of the PAS phenomenology that require interpretation.

## Background

In recent years there has been an explosion of interest in electrophysiological techniques that promote short-term changes in the excitability of human cerebral cortex, including patterned electrical or mechanical excitation of muscles and peripheral nerves, and methods of indirectly stimulating regions of the brain by means of transient magnetic fields or weak electrical currents. At least two motivations can be discerned. The first derives from the belief that interventions based on these techniques have the capacity to augment traditional neurorehabilitation practice, by promoting the physiological changes upon which recovery of function is based (e.g., Harris-Love and Cohen, 2006). The second is that such techniques provide means of studying brain plasticity at a systems level in humans (e.g., Muller-Dahlhaus et al., 2010).

In this context, Paired Associative Stimulation (PAS) has prominence both as a therapeutic intervention (e.g., Jayaram and Stinear, 2008; Castel-Lacanal et al., 2009), and as an experimental method with which to investigate Hebbian principles of synaptic plasticity. In the prototypical form of PAS (Stefan et al., 2000), a single electrical stimulus is directed to a peripheral nerve in advance of a magnetic stimulus delivered to the contralateral primary motor cortex (M1). The inter-stimulus interval is adjusted with a view to ensuring that inputs to M1 initiated by the afferent volley arising from the nerve stimulation occur simultaneously with the magnetic stimulation. Repeated pairing of the two sources of stimulation (i.e., association) over an extended period increases the excitability of corticospinal projections from M1. In circumstances in which the inter-stimulus interval is adjusted such that a corollary of the afferent volley may reach M1 after the magnetic stimulus, a decrease in corticospinal excitability has been reported (Wolters et al., 2003).

The neuroplastic adaptation revealed by PAS appears to exhibit several of the criteria designated for long-term potentiation (LTP) and long-term depression (LTD). Its effects evolve quickly, are reversible; and persist beyond the period of stimulation (McKay et al., 2002; Stefan et al., 2002). Pharmacological agents that interact with NMDA-receptor activity interfere with the outcomes of PAS, supporting the hypothesis that LTP-like changes are implicated (Stefan et al., 2002). In consideration of these properties, and assumptions that the alterations in excitability brought about by PAS were restricted to the cortical representations of muscles innervated by the peripheral nerve that was stimulated electrically, it has been suggested that PAS induced adaptation represents a form of associative LTP (and LTD) that is synapse-specific (Nitsche et al., 2007) and behaves in accordance with Hebbian principles (Stefan et al., 2000, 2004; Quartarone et al., 2003). More specifically, since the polarity of the induced effects appears contingent upon the order of the stimulus-generated cortical events, and the effective inter-stimulus intervals lie within a restricted (milliseconds) range, it has been proposed that the resemblance is to spike-timing dependent plasticity (STDP) (Muller-Dahlhaus et al., 2010).

Subsequent to the first report of this technique in 2000 by Stefan and colleagues, there have been a wide range of derivative investigations concerning, for example, the inter-stimulus intervals (ISIs) that are efficacious (e.g., Wolters et al., 2005; Kumpulainen et al., 2012), the muscles in which the effects can be elicited (e.g., Stefan et al., 2000; Stinear and Hornby, 2005; Carson et al., 2013), and variations in the extent to which they can be induced in various clinical populations (e.g., Castel-Lacanal et al., 2009; Monte-Silva et al., 2009; Bologna et al., 2012). Consideration has also been accorded to the levels of the neuraxis that are subject to influence by PAS (e.g., Stefan et al., 2000; Meunier et al., 2007; Di Lazzaro et al., 2009a,b; Russmann et al., 2009). As this corpus of work has accumulated, large inter-individual differences in response to PAS have been noted (e.g., Fratello et al., 2006). This has given rise to examination of such potential mediating factors as age (Fathi et al., 2010), cortical anatomy (Conde et al., 2012), and the role of specific genetic polymorphisms (Cheeran et al., 2008), among many others.

In view of the large body of empirical evidence that has accumulated, and particularly in light of the variations upon the original protocols that have been implemented, it is perhaps opportune to consider whether the phenomenology of PAS remains consistent with the cardinal features that were first disclosed. Any such assessment necessarily also has bearing upon interpretation of the effects of PAS in relation to specific cellular mechanisms, such as the expression of STDP.

## Scope of the review

This is an area of enquiry that is already extensive and burgeoning. In the present paper the focus will be maintained upon prototypical forms of PAS, in which stimulation of peripheral afferents is combined with single pulse transcranial magnetic stimulation (TMS) applied to contralateral M1. We pay particular attention to empirical observations that do not concur with standard assumptions, reasoning that these provide the necessary basis upon which to gauge the adequacy of current explanatory models. Consideration is not extended to studies in which PAS has been combined with other forms of non-invasive brain stimulation, for example in assessing the expression of homeostatic plasticity (e.g., Nitsche et al., 2007), or to the mediation of cognitive factors such as locus of attention (Stefan et al., 2004). In addition, the analysis is restricted to the motor system (cf. Schecklmann et al., 2011), and specifically to adaptations within higher brain centers (cf. Taylor and Martin, 2009; Cortes et al., 2011; Leukel et al., 2012).

Principally we characterize the effects of PAS in terms of changes in the excitability of projections from primary motor cortex—assessed through muscle responses evoked by TMS. These are brought about primarily by the trans-synaptic excitation of corticospinal cells. Although the amplitude of the motor-evoked potential (MEP) thus reflects the excitability of neurons in the motor cortex (Rothwell et al., 1991), it is also influenced by the state of the spinal motoneuron pool. While paired-pulse experiments may illuminate the contributory roles of microcircuits within M1, necessarily TMS-based techniques are unable to resolve changes in synaptic weights in the manner in which these are discriminable in reduced preparations (Verhoog et al., 2013).

## Timing dependency in PAS

In foundational reports (Wolters et al., 2003) it was noted that an increase in corticospinal excitability is achieved if the peripheral nerve stimulation is timed such that the initial phase of input to M1 arising as its corollary occurs synchronously with the delivery of a magnetic pulse over that area of cortex. If the relative timing is adjusted such that TMS is applied prior to the time at which a corollary of the peripheral afferent stimulation is anticipated to reach M1, repeated pairings may lead to a subsequent reduction in corticospinal excitability. Since the conclusion that PAS induced effects represent a distinct form of synapse-specific associative plasticity (i.e., STDP) is buttressed by the presence of timing dependency, the associated empirical findings demand particular attention.

### Upper limb muscles: excitatory effects

When the targets are projections to intrinsic hand muscles, the interval between the peripheral nerve stimulus and the TMS pulse is most commonly fixed (across participants) at 25 ms (“PAS25”), This protocol generates sustained increases in corticospinal excitability (e.g., Stefan et al., 2000; Wolters et al., 2003; Sale et al., 2007). It has also been shown that an ISI of 21.5 ms may have similar effects (Weise et al., 2006, 2011). Such increases can however also be obtained when a fixed inter-stimulus interval (ISI) of 35 ms is employed (Stefan et al., 2000).

On other occasions an individualized approach has been employed, whereby the latency of the N20 component of a somatosensory-evoked potential (SEP), elicited in each participant by stimulating the peripheral nerve, is used as a reference. In some instances the magnetic pulse has been timed to coincide with the N20 component (e.g., Ziemann et al., 2004). In other studies it has been delayed by 2 ms (“N20 + 2 PAS”) (e.g., Heidegger et al., 2010; Korchounov and Ziemann, 2011; Voytovych et al., 2012). In a recent investigation by Ilic and colleagues in which individual N20 latencies were used, this gave rise to ISIs ranging from 18.7 to 21 ms in a sample of 14 participants (Ilic et al., 2011). In this context, it is also worth noting that the effects of these protocols can vary markedly across participants, even when the ISI is determined on the basis of an individual's SEP. For example, Muller-Dahlhaus et al. (2008) noted that in a sample of twenty-seven people tested using a N20 plus 2 ms ISI, 14 showed the expected increase in corticospinal excitability, whereas the other thirteen exhibited a decrease (mean ratio post-PAS/pre-PAS = 1.00; range = 0.36–1.68). Kang et al. (2011) also failed to induce reliable changes in corticospinal excitability using a 25 ms ISI protocol (see also Fratello et al., 2006).

While for the most part the nature of the processes engaged by these different versions of the PAS protocol (Table 1) have not been subject to discrimination, it has been highlighted that the synaptic relays engaged at the latency of the N20 component may be distinct from those that are excited during intervals thereafter (Hamada et al., 2012). Indeed, excitatory effects induced using an ISI of 25 ms can be attenuated by the concurrent application of direct current stimulation to the cerebellum, whereas those brought about via an ISI of 21.5 ms appear to be unaffected by this manipulation (Hamada et al., 2012). A more general point is thereby illustrated. In seeking to appreciate the mechanistic basis of changes in corticospinal excitability instigated by PAS, consideration must necessarily be given to the presence of multiple neural pathways through which the constituent elements of this protocol are liable to exert their influence. With respect to projections to the muscles of the hand, the range of inter-stimulus (single nerve shock; single magnetic impulse) intervals for which excitatory effects can be obtained (18.7–35 ms) represents asynchronies at M1 well within the window necessary for the induction of LTP by STDP in reduced animal preparations (Bi and Poo, 1998; Dan and Poo, 2004, 2006). Nonetheless, this consistency does not in itself imply that a single mechanism is operative at all latencies within this range, or that the effects induced at any given latency are mediated principally by STDP.

**Table 1:** **Upper limb muscles: excitatory effects**.

**Muscle**	**Authors**	**ISI**	**Total number of stimuli**	**Stimulation period (mins)**	**Rate of delivery (Hz)**
APB	Fratello et al., 2006	25 ms	140 pairs	23	0.1
APB	Hamada et al., 2012	25/21.5 ms	180 pairs	15	0.2
APB	Heidegger et al., 2010	N20+2	90 pairs	30	0.05
APB	Ilic et al., 2011	N20	200 pairs	15	0.25
APB	Kang et al., 2011	25 ms	225 pairs	15	0.25
FCR	Kennedy and Carson, 2008	18.7 ms (mean)	84 pairs	28	0.05
			42 pairs	14	0.05
APB	Korchounov and Ziemann, 2011	N20+2	90 pairs	30	0.05
FCR	Meunier et al., 2007	20 ms	240 pairs	20	0.2
APB	Muller-Dahlhaus et al., 2008	N20+2	225 pairs	15	0.25
APB	Sale et al., 2007	25 ms	Short duration: 132 pairs	Short duration: 11	Short duration: 0.02
			Long duration: 90 pairs	Long duration: 30	Long duration: 0.05
APB	Stefan et al., 2000	25 ms	90 pairs	30	0.05
APB	Voytovych et al., 2012	N20+2	225 pairs	15	0.25
APB	Weise et al., 2006	21.5 ms	180 pairs	30	0.1
APB	Weise et al., 2011	21.5 ms	180 pairs	30	0.1
APB	Wolters et al., 2003	25 ms	90 pairs	30	0.05
APB	Wolters et al., 2005	N20	180 pairs	30	0.1
APB	Ziemann et al., 2004	N20	200 pairs	15	0.25

In a small number of cases the “classical” PAS protocols—in which a single peripheral afferent stimulus is delivered in association with a single pulse of TMS to the cortex, have been applied to study projections to muscles in the forearm. In these cases [in which the flexor carpi radialis (FCR) has typically been the focus of investigation] the ISI has either been fixed at 20 ms for all participants (Meunier et al., 2007), or determined through subtraction of the FCR M-wave onset latency from the MEP onset latency (to which 6 ms is added as an estimate of the time for the derivate of the afferent volley to travel from sensory to motor cortex). The resulting effects are however smaller than those observed for the intrinsic hand muscles, and in some cases they become clearly expressed only when there is additional cortical excitation promoted by contractions of homologous muscles of the opposite limb (Kennedy and Carson, 2008). We are not aware of attempts to examine the effect of changing ISIs for projections to the forearm muscles, however the intervals that have proved effective are consistent with those employed for muscles in the hand, given that the afferent volley traverses a shorter path (e.g., from a point of stimulation at the elbow) to higher brain centers.

### Upper limb muscles: inhibitory effects

In order to induce LTD-type effects in corticospinal projections to the hand (Table 2), it has been customary to employ a fixed ISI of 10 ms (“PAS10”), with a view to ensuring that a corollary of the afferent volley arrives at M1 after the magnetic cortical stimulus (e.g., Wolters et al., 2003; Monte-Silva et al., 2009; Thirugnanasambandam et al., 2011a,b; Weise et al., 2011). In a recent study however, Schabrun et al. (2013) reported that MEP amplitudes were reduced by a PAS protocol in which electrical stimulation of the median nerve was applied at fixed intervals of 250, 350, and 450 ms *following* the delivery of TMS to contralateral M1.

**Table 2:** **Upper limb muscles: inhibitory effects**.

**Muscle**	**Authors**	**ISI**	**Total number of stimuli**	**Stimulation period (mins)**	**Rate of delivery (Hz)**
FDI/APB	Amaya et al., 2010	N1-5 ms	200 pairs	13	0.25
APB	De Beaumont et al., 2012	10 ms	200 pairs	13	0.25
APB	Ilic et al., 2011	N20-5	200 pairs	15	0.25
APB	Kang et al., 2011	10 ms	225 pairs	15	0.25
ADM	Monte-Silva et al., 2009	10 ms	90 pairs	30	0.05
APB	Muller et al., 2007	N20-5	225 pairs	15	0.25
FDI	Potter-Nerger et al., 2009	N20-5	200 pairs	15	0.25
APB	Rajji et al., 2011	10 ms	180 pairs	30	0.1
APB	Schabrun et al., 2013	250, 350,450 ms following TMS	90 pairs	30	0.05
ADM	Thirugnanasambandam et al., 2011a	10 ms	90 pairs	30	0.05
ADM	Thirugnanasambandam et al., 2011b	10 ms	90 pairs	30	0.05
APB	Voytovych et al., 2012	N20-5	225 pairs	15	0.25
APB	Weise et al., 2006	10 ms	180 pairs	30	0.1
APB	Weise et al., 2011	10 ms	180 pairs	30	0.1
APB	Wolters et al., 2003	10 ms	90 pairs	30	0.05
APB	Ziemann et al., 2004	N20-5	200 pairs	15	0.25

In several other investigations individual ISIs have been calculated by means of the SEP N20 latency (e.g., Ziemann et al., 2004; Muller et al., 2007; Potter-Nerger et al., 2009; Ilic et al., 2011; Voytovych et al., 2012). The ISIs calculated by Ilic et al. (2011) on this basis (i.e., N20 latency minus 5 ms) yielded values longer than the conventional 10 ms interval used to induce inhibition (13.7–16 ms).

A number of investigators have however failed to obtain consistent reductions of corticospinal excitability following administration of a PAS10 protocol (e.g., Kang et al., 2011; Rajji et al., 2011). Weise et al. (2006), recorded a reliable reduction in APB MEP amplitudes at 45–55 min, but not at five other time points following the intervention.

In the only study of which we are aware that has been conducted in non-human primates, Amaya et al. (2010) applied 13 min of PAS to two awake trained rhesus monkeys. On the basis of an estimate of 12 ms for the latency of the N1 component of the SEP generated by contralateral median nerve stimulation, ISIs of 5 and 15 ms were employed (analogous to the PAS-10 and PAS-25 protocols used in humans). Whereas PAS based on an ISI of 15 ms led to reliable facilitation of MEP amplitude (265% of baseline) during a 2 h period following the intervention, no changes in corticospinal excitability were obtained when an ISI of 5 ms was used.

### Lower limb muscles

PAS protocols (Table 3) are also capable of inducing changes in the excitability of corticospinal projections to the muscles of the lower limb (Uy et al., 2003; Stinear and Hornby, 2005; Mrachacz-Kersting et al., 2007; Kumpulainen et al., 2012). In a study in which common peroneal nerve (CPN) stimulation and bilateral TMS were paired during treadmill walking, an ISI equivalent to the estimated MEP latency for the tibialis anterior (TA) muscle plus 5 ms was employed with a view to producing LTP-like effects. This ISI was gauged to result in a corollary of the CPN stimulation reaching M1 no more than 10 ms prior to TMS (during late swing around heel strike). In a further condition, the ISI was 10 ms shorter than the estimated MEP latency—judged to have ensured that the TMS was delivered prior to the corollary of the peripheral volley arriving in cortex (Stinear and Hornby, 2005). The excitability of corticospinal projections to TA (obtained during the late swing phase of walking prior to and following the 10 min intervention) was increased by the first protocol, and diminished by the second protocol. It emerges however that an ISI (MEP latency + 5 ms) that induces facilitation when the stimulus pairs are delivered during the late swing phase, leads to inhibition of the projections to TA when the PAS is administered during mid swing. Indeed, facilitation could only be induced using this ISI when the application occurred in a narrow time window against a background of voluntary EMG activity in TA (Prior and Stinear, 2006). Nonetheless, it also appears possible to obtain facilitation (assessed during subsequent walking) when this ISI is used at rest, although there is a dependency upon the intensity of the magnetic stimulus (Jayaram et al., 2007). Corresponding inhibitory effects (assessed during walking) have been obtained when PAS is administered at rest using an ISI 8 ms shorter than the estimated MEP latency (Jayaram and Stinear, 2008).

**Table 3:** **Lower limb muscles**.

**Muscle**	**Authors**	**ISI**	**Total number of stimuli**	**Stimulation period (mins)**	**Rate of delivery (Hz)**
TA	Jayaram and Stinear, 2008	MEP latency −8 ms	120 pairs	4	0.5
TA	Jayaram et al., 2007	MEP latency +5 ms	120 pairs	4	0.5
SOL	Kumpulainen et al., 2012	6, 12, 18, and 24 ms	200 pairs	Variable	0.2
TA	Mrachacz-Kersting et al., 2007	20, 30, 40, 45, 50, 55, 60 ms	360 pairs	30	0.2
TA/SOL	Prior and Stinear, 2006	MEP latency +5 ms	120 pairs	10	0.2
TA	Roy et al., 2007	15–90 ms after TMS	90 pairs	15	0.1
TA/SOL	Stinear and Hornby, 2005	MEP latency +5	120 pairs	10	0.2
		MEP latency −10			
TA	Uy et al., 2003	35 ms	180 pairs	30	0.1

Mrachacz-Kersting et al. (2007) also demonstrated that the effects of PAS directed at the projections to TA were accentuated markedly when the pairing of electrical stimulation of the CPN (at motor threshold) and bilateral magnetic stimulation of M1 was delivered during dorsi-flexion contractions [~5–10% maximum voluntary contraction (MVC)]. On the basis of SEP recordings (N34 peak), it was estimated that the corollary of the afferent volley reached M1 46–57 ms poststimulation. In this context, ISIs of 45, 50, and 55 ms yielded facilitation. In contrast, an ISI of 40 ms—TMS in advance of the estimated arrival of afferent evoked volley at M1, decreased the amplitude of MEPs elicited in TA. Notably however, facilitation of corticospinal projections to TA can also be obtained using ISIs tailored to achieve arrival of sensory mediated inputs to M1 over a range of 15–90 ms *following* cortical stimulation (Roy et al., 2007).

When projections to the soleus (SOL) muscle is the focus of investigation (Kumpulainen et al., 2012), and the first negative peak (P32) of the lower limb SEP (corresponding to the N20 component of the median nerve SEP) is used as a reference, reliable increases in corticospinal excitability have been obtained using an ISI of the P32 latency plus 18 ms. No such changes were registered for ISIs corresponding to the P32 plus 12 or plus 24 ms. A decrease in MEP amplitude was however reported when an ISI of P32 plus 6 ms was employed.

In summary, although the number of completed studies remains relatively small, it is apparent that the range of ISIs that is effective in inducing the facilitation of corticospinal projections to muscles of the lower limb is wider than that employed customarily in experiments on the upper limb, and beyond the upper boundary of intervals used to examine STDP in reduced preparations (e.g., Table 1 of Dan and Poo, 2006). Critically, in this context potentiation of corticospinal output can be achieved using PAS protocols that are likely to result in a corollary of the peripheral afferent volley reaching M1 after magnetic stimulation applied to the same brain region (Roy et al., 2007). Furthermore, the effects of these interventions are generally accentuated when there is additional cortical excitation associated with background contraction of the target muscle (Prior and Stinear, 2006; Mrachacz-Kersting et al., 2007). It has been noted that the physiological effects of (bilateral) magnetic stimulation applied using large double cone coils may differ from those arising from the impulses applied to cortical representations of hand muscles, particularly with respect to the relative contribution of I1 and later waves (Di Lazzaro et al., 2001). In addition, the excitability of M1 circuits projecting to leg muscles appears to be more readily modified by (electrical) peripheral afferent stimulation than those of the intrinsic hand muscles (Roy et al., 2007). These qualifications serve to highlight the limitations of using phenomenology alone as a basis upon which to infer mechanism. More specifically, there exist variants of PAS for which the associated effects fail to exhibit some of the cardinal features upon which attributions of mechanism have previously been based.

### Trains of stimulation

While with respect to the upper limb, investigations employing single pulse peripheral nerve stimulation appear to corroborate the assumption that the precise inter stimulus interval is critical in determining the nature of PAS induced effects, somewhat different conclusions may be drawn on the basis of experiments in which trains of afferent stimulation have been utilized. In several studies focusing on the state of corticospinal projections to hand and forearm muscles in healthy adults, trains of 500 ms duration consisting of 1 ms square waves delivered at 10 Hz (i.e., 5 stimuli per train) have been employed (Ridding and Taylor, 2001; McKay et al., 2002; Castel-Lacanal et al., 2007; Carson et al., 2013). In a seminal study in which the peripheral stimulation was applied over the motor point of FDI, Ridding and Taylor administered TMS stimuli 25 ms after the *onset* of each train. Following a 30 min intervention, substantial [200 ± 153% (SD)] increases in the amplitude of MEPs elicited in FDI were reported. Using the same protocol, comparable results were reported by McKay et al. (2002). When the TMS is administered 25 ms following the *last* shock of the train, effects of a similar nature are obtained when either the ECR (Castel-Lacanal et al., 2007) or the FCR (Carson et al., 2013) motor point are in receipt of stimulation. In the two variants of the train protocol therefore, there is a disparity of 50 ms with respect to the relative timing of the magnetic stimulus and the proximate peripheral shock. Yet both variants appear effective in potentiating the excitability of descending projections to the target muscle.

Equivalent outcomes were reported when the method introduced by Castel-Lacanal et al. (2007) was applied in stroke survivors, both early in the recovery phase and at 1-year post injury (Castel-Lacanal et al., 2009). In other circumstances in which both the peripheral nerve stimulation and TMS has been applied at 5 Hz over a 2 min interval, increases in the excitability of projections to the APB muscle were obtained if each TMS pulse was delayed by 25 ms with respect to the preceding peripheral (median) nerve stimulus. Reliable changes in corticospinal excitability were not however expressed if the delay was set at 10 ms (Quartarone et al., 2006). In the two cases of which we are aware, PAS protocols based on trains of electrical stimulation applied to the CPN have given rise to weak effects on the excitability of projections to TA that were not expressed consistently within samples of healthy young adults (Perez et al., 2003) or older stroke survivors (Uy et al., 2003).

### A reflection upon timing dependency

In PAS protocols in which a single shock is applied to a peripheral nerve in the upper limb in close temporal contiguity (<35 ms) with a magnetic pulse delivered over the contralateral hemisphere, the order in which the physiological sequelae exert their effects upon neural circuits within M1 (when paired repeatedly), determines the polarity of the changes in corticospinal excitability that follow. If the corollary of the ascending afferent volley is in advance of excitation arising from TMS, potentiation tends to occur. If the sequence of these events is reversed, inhibition is more likely.

On the basis of the most common PAS variants alone, it is tempting to conclude not only that the order of the stimulus-generated cortical events is critical, but also that the effective inter-stimulus intervals lie within a very restricted range. If however consideration is extended to other contexts in which PAS has been employed, a somewhat different set of inferences is likely to be drawn. This is due to the fact that with respect to projections to the lower limb, PAS protocols that result in a corollary of the peripheral afferent volley reaching M1 tens of milliseconds after the application of TMS result in sustained increases in excitability. Furthermore, when trains of electrical stimulation are applied to the upper limb, the ISIs that are effective in potentiating the corticospinal response extend over a span of at least 50 ms.

As there is a paucity of studies in which ranges of inter-stimulus intervals have been varied systematically, particularly for target muscles in the upper limb, it is not possible to offer definitive conclusions concerning those that might prove effective in inducing facilitation or inhibition of corticospinal projections. As such, some of these questions remain open. Wolters et al. (2003) assessed ISIs of −10, 0, 5, 10, 15, 20, 25, 35, and 50 ms. Although reliable facilitation was seen only at 25 ms and reliable inhibition only at an ISI of −10 ms, intervals greater than 20 ms tended to produce facilitation, whereas ISIs of 0, 5, and 10 ms tended to produce inhibition. Weise et al. (2013) used ISIs adjusted to the N20 latency (i.e., N20 – ISI) of 5.5, 7.0, 8.5, 10, and 11.5 ms, and observed that inhibition of APB could be obtained at adjusted ISIs of 8.5 and 10 ms. Dileone et al. (2010) reported on the basis of a sample of five participants that no changes in MEP amplitude were induced by an ISI of 100 ms. A similar observation was made by Kang et al. (2011) in the context of an investigation in which ISIs of 10 and 25 ms were similarly ineffective. There is certainly considerable variability across individuals. In some people an ISI of 25 ms can depress MEP amplitude, whereas an ISI of 10 ms has a potentiating effect (Huber et al., 2008). In light of the range of inter-stimulus intervals that have proved to be effective in studies of the lower limb, and when trains of peripheral nerve stimulation are applied to the upper limb, the possibility remains that the upper boundary of that range is beyond that which is characteristic of STDP—as studied in reduced preparations. We will return to this issue in some of the sections that follow.

## Muscle specificity of PAS induced effects

It has frequently been proposed that PAS induced adaptation represents a form of neuroplastic modification that is synapse-specific (e.g., Nitsche et al., 2007). In this regard, the term “topographical specificity” (e.g., Morgante et al., 2006; Castel-Lacanal et al., 2007; Quartarone et al., 2008) has been used to imply that alterations in excitability brought about by PAS are restricted to the cortical representations of muscles innervated by the peripheral nerve that was stimulated electrically (Stefan et al., 2000). The empirical origins of these suppositions are however difficult to discern. In this section we assess the degree to which the extant literature supports the notion of topographical (i.e., muscle) specificity.

### Upper limb muscles: unitary peripheral stimulus

In many studies in which PAS protocols are employed, EMG recordings are obtained only from a single (target) muscle. This is typically either the ulnar nerve innervated abductor digiti minimi (ADM), the median nerve innervated abductor pollicis brevis (APB), or the ulnar nerve innervated first dorsal interosseus (FDI). In some cases however potentials evoked in other muscles are recorded prior to and following the administration of PAS. For example, in the seminal study by Stefan et al. (2000), the median nerve was stimulated electrically (at the level of the wrist), and although APB was the primary focus of interest, MEPs were also recorded from ADM and the musculocutaneous nerve innervated biceps brachii (BB) muscle. PAS induced increases in the amplitude of MEPs recorded in each of these three muscles. Although the magnitude of the effect was larger for APB than for BB, the changes registered for the ulnar nerve innervated ADM were not distinguished from those obtained for APB—which is innervated by the nerve that received the electrical stimulation (p. 577). In at least one instance this protocol has yielded effects that are markedly larger for ADM than for APB (Cheeran et al., 2008).

Using precisely the same intervention, Quartarone et al. (2003) reported that for healthy adults, increases in the amplitudes of MEPs recorded in the ulnar nerve innervated FDI were of comparable effect size to those obtained for the (target) APB (see also Rosenkranz and Rothwell, 2006; cf. Quartarone et al., 2008; Elahi et al., 2012). Notably, Potter-Nerger et al. (2009) demonstrated elevations in the amplitude of MEPs recorded from the ulnar nerve innervated FDI, using a median nerve stimulation PAS protocol, and a similar trend for ADM, in the absence of corresponding changes for the APB muscle (see supplementary figure S1). Employing a variation of the Stefan et al. protocol in which the peripheral electrical stimulation was applied to the ulnar nerve at the wrist, Dileone et al. (2010) reported increases in the excitability of corticospinal projections to the target FDI and the median nerve innervated APB, although the latter were most prominent immediately following the cessation of the intervention. In other cases in which the changes in the excitability of corticospinal projections to non-target muscles have not been statistically reliable, the effects have consistently been in the same direction as those induced in the target muscle (e.g., APB target—ADM comparison: Fratello et al., 2006; Morgante et al., 2006; Weise et al., 2011; Popa et al., 2013; APB target—FDI comparison: Quartarone et al., 2009; ADM target—APB comparison: Weise et al., 2006).

Notably, the limited number of studies in which MEPs have been obtained for multiple muscles prior to and following the administration of 10 ms ISI PAS protocols also reveal changes in the excitability of corticospinal projections to muscles (in the hand) which are innervated by peripheral nerves other than the one that is the target of the electrical stimulation. Specifically, Weise et al. (2006, 2011) reported that when the median nerve was stimulated, 10 ms ISI PAS gave rise to decreases in MEP amplitude for the median nerve innervated APB, and increases for the ulnar nerve innervated ADM (see also Weise et al., 2013). Whereas, using a protocol in which TMS was delivered 5 ms in advance of the individual N20 latency of the median nerve SEP, Potter-Nerger et al. (2009) decreases in MEP amplitude were obtained both for the target APB and for the ulnar nerve innervated FDI (see supplementary figure S1).

### Upper limb muscles: trains of peripheral stimulation

When trains of peripheral stimulation are employed, the distributed nature of the effect does not appear to be contingent upon the specific muscle that is the target of the stimulation. Castel-Lacanal et al. (2007) applied PAS comprising 10 Hz (500 ms) trains of electrical stimulation to the ECR motor point (and single pulse TMS), and obtained increases in the magnitude of MEPs that corresponded to large effect sizes for both ECR (eta-squared = 0.27) and FCR (eta-squared = 0.26). Ridding and Taylor (2001) induced a mean increase of 128 ± 132% (SD) in the excitability of corticospinal projections to the median nerve innervated FCR, by means of PAS applied to FDI. Employing the same stimulation protocol, and recording MEPs in ADM and APB, McKay et al. (2002) noted that the increases in corticospinal excitability obtained for FDI were expressed similarly for ADM. A corresponding trend was also apparent for the APB muscle.

Carson et al. (2013) demonstrated that when trains of electrical stimulation were applied to the musculocutaneous nerve innervated BB, the effects of PAS were also expressed in FCR, and in ECR—which is innervated by the radial nerve. When the FCR was the target, increases in the excitability of corticospinal projections to BB and ECR were obtained—in addition to those present for FCR. No impact of either BB or FCR focused PAS was apparent for projections to the lateral head of triceps brachii, which shares with ECR the property of innervation by the radial nerve. In contrast, Quartarone et al. (2006) reported no distributed effects in FDI and ECR, when TMS was delivered over the APB “motor hot spot” and the median nerve was stimulated at the wrist.

### Lower limb muscles

Assessing somatotopy in relation to lower limb muscles is complicated by the use in many instances of background contractions as elements of the induction protocol. These necessarily give rise to patterns of facilitation and (e.g., antagonist) inhibition, the effects of which cannot easily be dissociated from those of the PAS. When applied during treadmill walking for example, cycle-phase-specific facilitation of TA arising from electrical stimulation of the CPN paired with TMS, also results in the suppression of MEPs recorded from semimembranosus (SM)—which is innervated by the tibial nerve (TN; Prior and Stinear, 2006). Using a (treadmill walking) PAS protocol designed to decrease the excitability of projections to TA, Stinear and Hornby (2005) reported increases in the area of MEPs recorded to SOL. Using ES delivered to the CPN, and TMS latencies determined from the N34 peak, Mrachacz-Kersting et al. (2007) reported that increases in the amplitude of TA MEPs arising from PAS delivered during weak (~5–10% MVC) dorsiflexion, were not accompanied by similar changes for SOL.

Employing ISIs designed to achieve arrival of CPN stimulation generated inputs to M1 over a range of 15–90 ms following TMS, Roy et al. (2007) observed that when PAS was delivered with the muscles quiescent, increases in the excitability of corticospinal projections were obtained not only for the target TA muscle, but also for the homologous muscle of the opposite limb. In a related context, Roy and Gorassini (2008) reported that electrical stimulation of the TN at the ankle and the posterior tibial nerve (PTN) at the knee had strong facilitatory effects on MEPs at latencies a few milliseconds after the arrival of afferent inputs at the somatosensory cortex, and that these effects were both non-specific and diffuse. Stimulation of TN at the ankle, for example, had “homotopic” (occurring at the corresponding part of the body) effects on projections to abductor hallucis (AH) and “heterotopic” effects on those to TA (see also Uy et al., 2003). Using a PAS protocol in which stimulation was delivered to the TN at the popliteal fossa, Kumpulainen et al. (2012) obtained increases in the excitability of corticospinal projections to SOL, but did not report (“*P* > 0.05”) similar outcomes for TA.

### A reflection upon muscle specificity

Contrary to received wisdom the empirical evidence indicates that restriction of the effects of PAS to muscles innervated by the peripheral nerve in receipt of electrical stimulation is the exception rather than the rule. Furthermore, there are several reported instances in which changes in the excitability of corticospinal projections induced by classic PAS protocols have been more pronounced for muscles that are innervated by a different nerve (e.g., Cheeran et al., 2008; Potter-Nerger et al., 2009). Indeed, given effects obtained for the ulnar nerve innervated ADM that could not be distinguished from those obtained for APB (innervated by the median nerve that received the electrical stimulation), Stefan et al. (2000) referred in their formative paper to a “somatotopic gradient.” The point that the muscle specificity of the changes in corticospinal excitability brought about by PAS is relative rather than absolute, has also been made by other commentators (e.g., Quartarone et al., 2003). In some of the sections that follow we will give further consideration to mechanisms via which somatotopic gradients might emerge.

## Neural circuits through which the effects of PAS are manifested

### Cortical

Paired pulse TMS is a tool widely used to investigate inhibitory and facilitatory circuits in the human cerebral cortex (Ortu et al., 2008). The technique involves delivery of a conditioning stimulus (s1) and a test stimulus (s2) through the same coil, with the ISI and the intensities of the two pulse being adjusted in a manner appropriate for investigation of the interneuronal circuits that are the focus of interest (Kujirai et al., 1993; Alle et al., 2009; Wagle-Shukla et al., 2009). In regards to PAS, the phenomena that have been investigated by this means include short interval intracortical inhibition (SICI) and long interval intracortical inhibition (LICI) (Kujirai et al., 1993), and intracortical facilitation (ICF).

### Short interval intracortical inhibition (SICI)

The term SICI reflects the elicitation of a response to the test stimulus that is diminished in size when it is preceded by a conditioning stimulus—at intervals typically ranging between 1 and 5 ms. It is thought that the cellular processes underlying this effect are mediated, at least in part, by GABA^a^ receptors (Di Lazzaro et al., 2006; Peurala et al., 2008). While it is an oversimplification to consider changes in SICI simply as an index of GABA^a^ activity—since there is little direct evidence for this association in humans, benzodiazapines, which are positive modulators of GABA^a^, receptor function enhance SICI (Di Lazzaro et al., 2001). In contrast, GABA reuptake inhibitors decrease levels of SICI (Werhahn et al., 1999; Ziemann, 2004). The potential impact that PAS may have upon intracortical circuits mediating the expression of SICI has been investigated in a large number of studies. By and large these have failed to yield consistent changes in SICI following the administration of PAS25 protocols for which an intrinsic hand muscle is the target (Stefan et al., 2002; Quartarone et al., 2003; Rosenkranz and Rothwell, 2006; Sale et al., 2007, 2008; Cirillo et al., 2009; Russmann et al., 2009; Di Lazzaro et al., 2011; Elahi et al., 2012; Schabrun et al., 2013). To some degree this may reflect the fact that the expression of SICI is highly variable both within and between individuals (Wassermann, 2002). While there is very little evidence to indicate that PAS25 has a reliable effect on the manifestation of SICI (see also Ridding and Taylor, 2001; Castel-Lacanal et al., 2007; Roy et al., 2007), this does not preclude the possibility that the efficacy of the intervention is influenced by the state of the inter-neuronal networks to which the SICI technique is sensitive (Ridding and Flavel, 2006). Consistent with this hypothesis, Elahi et al. (2012) demonstrated that when SICI is evoked simultaneously with the administration of a PAS25 protocol, the usual facilitation of corticospinal excitability is not obtained. With respect to PAS10 protocols, we are aware of only two studies in which SICI has been monitored in conjunction with this variant. Both Russmann et al. (2009) and Di Lazzaro et al. (2011) reported decreases in this measure of intracortical inhibition as a result of the intervention.

### Long interval intra-cortical inhibition (LICI)

Long interval intra-cortical inhibition (LICI) is measured at ISIs between 50 and 200 ms. It is putatively mediated by GABA^b^ receptors (Werhahn et al., 1999; McDonnell et al., 2006). While the effects of a facilitating PAS protocol (N20+2) are blunted by the prior administration of Baclofen (BAC)—a selective GABA^b^ receptor agonist, it does not necessarily follow that the state of cortical circuits sampled by the LICI technique will be altered by its administration. Meunier et al. (2012) did however observe that LICI decreased when afferent stimulation was paired (25 ms ISI) with “low intensity” TMS (evoking a MEP of 0.5 mV), but not when an intensity of TMS sufficient to generate an MEP of 1 mV in the target FPB muscle was used. Similarly, Russmann et al. (2009) reported that LICI was reduced by administration of a PAS25 protocol (evoking a MEP of 0.5 mV in FPB), and increased transiently by a PAS10 variant. De Beaumont et al. (2012) found no significant changes in LICI arising from the application of a PAS10 intervention in which afferent stimuli were paired with TMS at an intensity that produced a MEP of 1 mV in the target APB. Notwithstanding other variations in protocol, on the basis of the small number of studies that have been completed, it appears that when the intensity of the cortical stimulus is moderate (leading to 0.5 mV MEPs in intrinsic hand muscles), PAS25 leads to a decrease in LICI, whereas PAS10 may cause an increase in LICI.

### Intracortical facilitation (ICF)

The term ICF refers to the elicitation of a response to the test stimulus that is increased in size when it is preceded by a conditioning stimulus—at intervals typically ranging between 7 and 20 ms, in the context of protocols similar to those used to elicit SICI (Kujirai et al., 1993; Ziemann et al., 1996). While it is believed that the net facilitation arises from a strong potentiating effect and a weaker inhibitory component (Hanajima et al., 1998; Hanajima and Ugawa, 2008), pharmacological studies that the dominant element is mediated by glutamatergic M-methyl-D-asparate (NMDA) receptors (Ziemann et al., 1998; Schwenkreis et al., 1999). As benzodiazepines also increase ICF however, a contribution of GABA^a^ receptors—expressed through the inhibitory component cannot be excluded (Ziemann, 2008). No changes in ICF have however been reported when PAS25 protocols have been employed, and hand muscles are the focus of interest (Di Lazzaro et al., 2011—15 ms ISI; Elahi et al., 2012 and Sale et al., 2007—10 ms ISI; Schabrun et al., 2013—13 ms). Similarly, no impact upon ICF has been observed when muscles in the lower limb (Roy et al., 2007) or the forearm (Castel-Lacanal et al., 2007) have been investigated. As Elahi et al. (2012) failed to demonstrate that ICF evoked simultaneously with the administration of a PAS25 protocol, exerted an impact upon the usual facilitation of corticospinal excitability, it can also be surmised that the efficacy of the intervention is insensitive to the state of the inter-neuronal networks sampled by the ICF technique.

### Short-interval intracortical facilitation (SICF)

It is also possible to obtain facilitation of a subthreshold test stimulus when a prior conditioning stimulus of threshold or suprathreshold intensity is delivered at discrete intervals of 1.0–1.5 ms, 2.5–3.0 ms and at ≈4.5 ms (Tokimura et al., 1996; Ilic et al., 2002). As the effect is not obtained when transcranial electrical stimulation (TES) is substituted for the magnetic stimulus, a M1 locus for what is termed short-interval intracortical facilitation (SICF) is presumed. In particular, as the effective ISIs are closely related to I-wave periodicity, an instrumental relationship is suspected (e.g., Hanajima and Ugawa, 2008). Benzodiazepines and barbiturates, which enhance the action of GABA^a^ receptors, attenuate SICF (Ziemann et al., 1998; Ilic et al., 2002), whereas the NMDA receptor antagonist memantine does not alter the effect. To the best of our knowledge, the impact of PAS upon SICF has been investigated in only one instance. Ridding and Taylor (2001) reported that SICF increased at short ISIs (0.8–1.7 ms) following administration of a protocol that comprised trains of afferent stimulation.

### Cortical silent period (CSP)

Following the elicitation of a MEP in a contracting peripheral muscle, there occurs a period of EMG silence. While spinal circuitry may be implicated in the early (~50 ms) part of the silent period, the subsequent portion appears to be due to processes operating at the level of the cerebral cortex (Wilson et al., 1993; Ziemann et al., 1993; Brasil-Neto et al., 1995; Chen et al., 1999b; Tergau et al., 1999). The duration of the cortical silent period (CSP) is influenced to a greater degree by TMS intensity than level of muscle contraction (Kojima et al., 2013). As it shares this property with the degree of SICI induced by TMS (i.e., CS intensity), which is not the case for ICF, it has been proposed that the CSP duration is governed by the state of inhibitory interneurons within M1 that also mediate the expression of SICI (Kojima et al., 2013). On the basis of a review of pharmacological interventions it has been suggested previously (Ziemann, 2004) that, as with LICI, the duration of the late part of the CSP is mediated by GABA^b^ receptors. An elongation of CSP duration following the administration of PAS25 protocols has been reported on numerous occasions (Stefan et al., 2000, 2004; Quartarone et al., 2003; Sale et al., 2007, 2008; Cirillo et al., 2009; De Beaumont et al., 2012; Elahi et al., 2012; cf. Di Lazzaro et al., 2011). In the single study in which this measure has been used to examine a PAS protocol that utilizes trains of afferent stimulation (Ridding and Taylor, 2001), no such prolongation was obtained. In addition, it appears that duration of the CSP is not influenced by a PAS10 protocol (Di Lazzaro et al., 2011; De Beaumont et al., 2012) or a N20-5 protocol (Potter-Nerger et al., 2009).

### Short afferent inhibition (SAI)

The term short afferent inhibition (SAI) refers to the diminution of MEP amplitude that occurs following administration of a prior conditioning afferent stimulus (typically 0.2–1 ms duration at an intensity 2—3 times perceptual threshold or that which evokes a visible twitch in the target muscle) applied to a peripheral nerve. The latency at which the effect is most prominent is 13–19 ms when forearm muscles (FCR and ECR) are the focus of interest, and the nerve is stimulated at the level of the elbow (Bertolasi et al., 1998), and ~20 ms when hand muscles (i.e., FDI and APB) are under investigation and nerve (i.e., median) stimulation is applied at the wrist (Tokimura et al., 2000). It is thought that the effect is produced by modulation of the I2 and I3 waves of the descending corticospinal volley (Tokimura et al., 2000). As scopolamine (an Ach antagonist) reduces SAI, but does not exert a similar influence on SICI, distinct mediating neural circuits are presumed (Di Lazzaro et al., 2000). In addition, the benzodiazepine lorazepam increases SICI, but decreases SAI (Di Lazzaro et al., 2005). Electrophysiological studies of the interactions between SICI and SAI further suggest that these phenomena are expressed via the influence of distinct, but convergent and reciprocally connected, GABAergic inhibitory interneurons that project onto corticospinal neurons (Alle et al., 2009). When MEPs are recorded during the administration of PAS, they are attenuated initially (with respect to pre-intervention controls), most likely as a consequence of SAI type effects. This effect declines through the time course of the induction period (e.g., Di Lazzaro et al., 2011; Elahi et al., 2012; Hamada et al., 2012), presumably due to the overall increase in the excitability of the corticospinal projections brought about by the intervention. When however the amplitude of the MEP obtained following the conditioning afferent stimulus is normalized with respect to the amplitude of a test stimulus alone, no changes in SAI are seen to occur as a result of conventional PAS25 protocols (Stefan et al., 2002; Di Lazzaro et al., 2011; Elahi et al., 2012; Hamada et al., 2012; Schabrun et al., 2013). In this respect therefore, SAI mirrors SICI. The two measures do however diverge in so much as no change in SAI has been reported following PAS10 (Di Lazzaro et al., 2011), whereas in this context a decrease in SICI is obtained (Russmann et al., 2009; Di Lazzaro et al., 2011).

### Long afferent inhibition (LAI)

The attenuation of MEP amplitude that is also obtained when the interval between the peripheral afferent stimulation and the subsequent TMS is in the region of 200 ms is referred to as long-latency afferent inhibition (Sailer et al., 2002, 2003). As the amplitude of the F-wave evoked by supramaximal stimulation of the peripheral nerve is not reduced at a conditioning-test interval of 200 ms, the post-synaptic state of spinal motoneurons is not believed to be a principal determinant (Chen et al., 1999a). A contribution of cortical structures in addition to the primary sensory and motor areas, and of sub-cortical elements, to the expression of LAI cannot however be excluded (Classen et al., 2000; Sailer et al., 2003, 2007). On the basis of observations that LAI interacts with (inhibits) LICI, it has been inferred that there is some degree of shared mediation by GABA^b^ receptors (Sailer et al., 2002), however the neurotransmitters involved in LAI have not yet been corroborated using pharmacological approaches (Ni et al., 2011). Using a PAS25 protocol based on “low intensity” TMS (evoking a MEP of 0.5 mV), Meunier et al. (2012) reported immediate and sustained decreases in LAI (150 ms ISI) evoked for projections to the target FPB—an effect that was broadly similar to that expressed for LICI. No such changes were obtained when an intensity of TMS sufficient to generate an MEP of 1 mV in the target FPB muscle was used in the delivery of PAS. Consistent with these outcomes, Russmann et al. (2009) demonstrated a reduction in LAI (150 ms ISI) evoked in FPB that followed the time course of decreases in LICI induced by a PAS25 (low intensity TMS) protocol (there was no consistent change attributable to PAS10). In contrast, marked increases in LAI (240 ms ISI) were observed following PAS25, whereas decreases were seen following PAS10 (Russmann et al., 2009).

### Spinal

In the small number of studies that have sought to examine potential changes in excitability at the level of the spinal cord following PAS, F-waves have most commonly been obtained, even though this technique has characteristics that limit its effectiveness as a test of spinal motoneuron excitability, a problem that is particular acute when comparisons are drawn with responses evoked by cortical magnetic stimulation (Carson et al., 2004; Taylor, 2006). Investigations utilizing this approach have generally failed to obtain indications of changes in spinal motoneuron excitability following PAS (Stefan et al., 2000; Wolters et al., 2003; Quartarone et al., 2006; Meunier et al., 2007; Mrachacz-Kersting et al., 2007; Thabit et al., 2010). Converging findings have however been derived using electrical transmastoid (cervicomedullary) stimulation, which activates corticospinal axons below the level of the cortex (Ugawa et al., 1991; Taylor et al., 2002). As this technique is uncomfortable it has been used sparingly in PAS studies, and in each case a very small number of participants has been assessed (Stefan et al., 2000; McKay et al., 2002; Wolters et al., 2003).

Employing FCR as the target muscle, and using a PAS protocol in which median nerve stimulation was paired with TMS, Meunier et al. (2007), reported that changes in the slope of the H reflex recruitment curve occurred in parallel with intervention induced increases in corticospinal excitability. A similar pattern was obtained in the small number of participants from whom H-reflexes could be elicited in APB, when a PAS25 protocol was used with this muscle as the target. In a follow up study, it was demonstrated that the PAS-induced change in the H-reflex is mediated by a decrease of presynaptic Ia inhibition of FCR terminals (Lamy et al., 2010). On the basis of the evidence currently available, it is not possible to resolve whether this effect is contingent upon alteration of descending inputs to presynaptic interneurons acting on the Ia pathway, or changes in presynaptic networks at the spinal level. It has been remarked that presynaptic [primary afferent depolarization (PAD)] interneurons, which receive extensive projections from Ia, Ib, and cutaneous afferents, may play an instrumental role in the latter regard (Lamy et al., 2010). It is also worth noting in this context that conventional PAS protocols (e.g., Stefan et al., 2000) employ a level of peripheral nerve stimulation (i.e., 3 × perceptual threshold) that is sufficient to elicit a contraction of the target muscle (Kennedy and Carson, 2008), and thus generate secondary reafference. The implications of this will be given further consideration in sections that follow. Roy et al. (2007) failed to obtain changes in the amplitude of H-reflexes recorded in TA, arising from a PAS protocol that induced increases in corticospinal excitability.

### A reflection on the expression of PAS-induced effects

The most direct source of evidence available in humans—that based on recording corticospinal volleys via electrodes implanted in the cervical epidural space (of 4 individuals), indicates that the PAS25 protocol does not alter the first wave of descending excitation generated by TMS given subsequently, but increases the amplitude of later waves (Di Lazzaro et al., 2009a). The complementary finding (from 2 individuals) is that the PAS10 protocol does not alter the first wave of descending excitation, but decreases the amplitude of later waves (Di Lazzaro et al., 2009b). In light of these results, and given indications that the post-synaptic state of spinal motoneurons is not altered by PAS, it is reasonable to conclude that the observed changes in corticospinal excitability are mediated principally at the level of the cortex. Is it also possible to resolve specific circuits within cortex that are implicated?

The summary conclusions that can be drawn from the studies described above are that (corticospinal) excitability enhancing PAS protocols (e.g., PAS25) do not alter expressions of SICI, ICF, or SAI. They do however elongate the CSP, and may decrease LICI and LAI (when the peripheral stimulation applied during PAS is paired with low intensity TMS). Inhibitory protocols (e.g., PAS10) have been investigated less thoroughly. As a consequence, it is possible to surmise only that they tend to decrease SICI, and have no apparent influence on the CSP.

On the basis of indications that TMS invoked silent periods were shortened by the delivery of (single pulse) high-intensity peripheral nerve stimulation over a range of intervals from 30 ms before to 70 ms after TMS (with the largest effect present at 20 ms before), Hess et al. (1999) concluded that the somatosensory input generated by the peripheral stimulation has privileged access to inhibitory interneuronal circuits within M1. In respect of observations that both the extent of LICI and the duration of the CSP increased with eliciting stimulus intensity, Hammond and Vallence (2007) proposed that the long-latency inhibitory circuits that mediate the LICI effect, are also those through which afferent feedback from the contracting muscle acts to modulate the time course of the silent period (see also Taylor et al., 1997; Thabit et al., 2010; Farzan et al., 2013). Given the equivalent pattern of variation that is obtained for LICI, LAI and the duration of the CSP, it might therefore be surmised that the state of these long latency inhibitory circuits is altered by facilitatory variants of PAS. Is it possible that the changes in late I-waves engendered by excitability enhancing forms of PAS reflect tonic modification of GABA^b^mediated projections operating via these circuits (e.g., Humeau et al., 2003).

With respect to LAI, it is notable that the measure itself does not exhibit muscle specificity. If the conditioning stimulus is applied to the median nerve at the wrist, in a fashion similar to that used in PAS protocols, the inhibition of MEP amplitude that is observed at an ISI of 200 ms is obtained not only for APB (median nerve innervated), but also for the FDI (Chen et al., 1999a; Abbruzzese et al., 2001), FCR (Abbruzzese et al., 2001), and ECR (Chen et al., 1999a) muscles. When an ISI of 100 ms is used, median nerve stimulation evokes equivalent levels of LAI in projections to APB, ADM, and FDI (Kotb et al., 2005). Similarly in relation to SAI, if the median nerve is stimulated at the wrist and an ISI ≈20 ms is employed, inhibition is obtained not only for APB, but also for FDI (Tokimura et al., 2000; Kotb et al., 2005; Devanne et al., 2009), ADM (Kotb et al., 2005), and ECR (Devanne et al., 2009). Median nerve stimulation at the antecubital fossa and radial nerve stimulation in the spiral groove each generate comparable SAI in projections to both FCR and ECR. If the ISI is defined in relation to the N20 component of the SEP, ISIs of N20, N2+2, and N20+4 elicit SAI in both FDI and APB when either the median and ulnar nerve are stimulated. In both cases, the level of inhibition is accentuated by increasing the intensity of afferent stimulation (Fischer and Orth, 2011). As such, with respect to both SAI and LAI there is a parallel with the lack of muscle specificity that characterizes the effects of PAS.

While consideration of the intracortical neural circuits that mediate the expression of phenomena such as SAI and LAI may provide insights in relation to those that are instrumental in relation to the effects of PAS, in any such assessment, it is necessary to maintain a conceptual distinction between circuits that may be necessary for the induction of changes in corticospinal output, but which are not altered functionally by the administration of PAS, and those that are modified acutely by PAS. In some but not necessarily all of these latter cases, the PAS induced changes may impact upon the excitability of descending corticospinal projections as registered through responses to TMS (i.e., at rest), or on voluntary motor output. For example, while it may be the case that a lack of muscle specificity is a characteristic shared by SAI, LAI and the effects of PAS, only the expression of long-latency afferent inhibition (LAI) but not that of SAI is altered by the intervention. Furthermore, although levels of SICI are not altered by PAS25, increases in corticospinal excitability normally induced by this protocol are blocked when SICI is evoked simultaneously with its administration (Elahi et al., 2012; see also Weise et al., 2013). In seeking to understand the roles played by specific circuits within cortex in mediating the effects of PAS, it would be extremely useful to have further interference studies of this type. To date however, pharmacological studies have provided the main source of evidence upon which to derive causal inferences, albeit at a systems level.

## Pharmacology of PAS-induced effects

As there are authoritative and comprehensive reviews dealing with the pharmacology of neuroplastic responses to non-invasive brain stimulation (Nitsche et al., 2012), and of cortical excitability measures (Ziemann, 2004, 2008; Paulus et al., 2008), we hereby provide only a summary pertinent to PAS that draws in part upon these previous works. Indeed, we explicitly adopt the structure of presentation of Nitsche et al. (2012)—conceiving of the glutamatergic system, voltage-gated ion channels and the GABAergic system as “drivers” of neuroplastic adaptation, and referring to the dopaminergic, cholinergic, serotonergic, and adrenergic systems as “modulators” of neuroplastic adaptation.

### The glutamatergic system—a driver of neuroplastic adaptation

As the N-methyl-d-aspartate (NMDA) receptor antagonist dextromethorphan (150 mg dose) blocks both the excitability enhancing effects of PAS25 (Stefan et al., 2002) and the excitability reducing effects of PAS10 (Wolters et al., 2003), a generalized dependence upon on NMDA receptor activation has been deduced. This drug is however also thought to act as a non-selective serotonin reuptake inhibitor, and as a sigma-1 receptor agonist with influence upon calcium signaling.

### Voltage-gated ion channels—a driver of neuroplastic adaptation

It has been reported that the voltage-gated sodium channel blocker lamotrigine tends to reduce the facilitating effect of a N20+2 PAS protocol (Heidegger et al., 2010). Nimodipine, which blocks L-type (long-lasting) voltage-gated calcium channels, eliminates the excitability reducing effects of PAS10 when given as a 30 mg dose (Wolters et al., 2003). It is thought that when applied chronically, but not acutely, in experimental systems, Gabapentin inhibits calcium currents through an influence on the trafficking of voltage-gated Ca^2+^ channels (Hendrich et al., 2008; but see also Eroglu et al., 2009). Administration of the drug (1100 mg) does not impact on the usually obtained effects of N20+2 PAS (Heidegger et al., 2010). Although the precise mode of action of the anticonvulsant levetiracetam has not always been clear, it is now believed that it inhibits voltage-gated Ca^2+^ channels (Vogl et al., 2012). A 3000 mg dose of this drug abolishes the increases in MEP amplitude otherwise induced by N20+2 PAS (Heidegger et al., 2010).

### The GABAergic system—a driver of neuroplastic adaptation

The facilitating effects of N20+2 PAS are blunted by 50 mg of the GABA^b^ receptor agonist baclofen (McDonnell et al., 2007). They are also diminished by administration of diazepam (20 mg)—a positive allosteric (binding to a specific subunit on the GABA^a^ receptor at a site distinct from the that of the endogenous GABA molecule) modulator of GABA (Heidegger et al., 2010). Tiagabine (25 mg) that is thought to act as a selective GABA reuptake inhibitor, permitting increased GABA availability for post-synaptic receptor binding, exerts a similar action (Heidegger et al., 2010). On the other hand, topiramate—having pharmacological properties that may include augmentation of GABA^a^ mediated inhibition (blockage of voltage-dependent sodium channels), has no such effects in 100 mg dosage (Heidegger et al., 2010).

### The dopaminergic system—a modulator of neuroplastic adaptation

Thirugnanasambandam et al. (2011b) delivered low (25 mg), medium (100 mg), or high (200 mg) doses of levodopa prior to PAS in 12 healthy volunteers. In low dose, levodopa abolished the usual effects of both PAS10 and PAS25 variants. In medium dosage, the induced effects were indistinguishable from those obtained in placebo conditions. At high dosage, the prior delivery of levodopa gave rise to an inhibitory influence of the PAS25 protocol on MEP amplitude, whereas the impact of PAS10 could not be differentiated from the placebo condition. This set of outcomes contrasts with the results of Kuo et al. (2008) who observed that a 100 mg dose of levodopa enhances the magnitude and duration of increases in corticospinal excitability induced by PAS25.

Administration of 400 mg of the selective dopamine D2 and D3 receptor antagonist sulpiride (with the intent of increasing the relative contribution of D1 receptors to dopaminergic activity) eliminates the inhibitory effects of PAS10, but has no impact upon increases in excitability brought about by PAS25. When however sulpiride (400 mg) was given in combination with 100 mg of levodopa, the inhibitory effect of PAS10 was preserved (and a typical profile of response to PAS25 obtained) (Nitsche et al., 2009). A 2 mg dose of the selective dopamine D2 receptor agonist Cabergoline does not appear to influence the excitability enhancing effects of N20+2 PAS (Korchounov and Ziemann, 2011). The D2 receptor agonist ropinirole exhibits an inverted “U”-shaped dose–response curve, whereby both high (1.0 mg) or low (0.125 mg) dosages of the drug impair the effects of a PAS25 protocol, whereas the attenuation exhibited following a medium dose (0.5 mg) is less pronounced. In contrast, ropinirole has no apparent impact upon the impact of PAS10 (Monte-Silva et al., 2009).

Haloperidol exhibits high affinity dopamine D2 receptor antagonism. When a 2.5 mg dose of the drug is given 2 h in advance of a N20+2 protocol, the usual facilitating effects of this intervention are not obtained (Korchounov and Ziemann, 2011). Methylphenidate acts primarily to inhibit the reuptake of dopamine and to a lesser extent norepinephrine, thus increasing the extracellular concentrations of these neurotransmitters. The prior delivery of 40 mg of this agent has no apparent impact upon the efficacy of N20+2 PAS (Korchounov and Ziemann, 2011).

### The cholinergic system—a modulator of neuroplastic adaptation

If the activity of the two major acetylcholine receptor subtypes [muscarinergic (mAChR) and nicotinergic (nAChR)] is promoted by administration of the cholinesterase inhibitor rivastigmine (3 mg), the positive impact on corticospinal excitability of PAS25 is enhanced relative to a placebo condition, between 20 and 30 min following the cessation of paired stimulation. The inhibitory effects of PAS10 are also accentuated, and particularly pronounced during a period from 25 min to 2 h post stimulation (Kuo et al., 2007). In contrast however, the cholinesterase inhibitor Tacrine (40 mg) does not appear to alter the effects of a N20+2 protocol (Korchounov and Ziemann, 2011). Using transdermal patches able to deliver 15 mg of nicotine (i.e., a nAChR receptor agonist) over 16 h, Thirugnanasambandam et al. (2011a) reported that when paired stimulation commenced 6 h following application of the patch, the effects of PAS25 were not distinguished from a placebo condition. On the other hand, the usual inhibitory influence of PAS10 was eliminated by the administration of nicotine. Biperiden is a M1 muscarinic receptor (mAChR) antagonist. When an 8 mg dose is delivered 2 h before N20+2 PAS, there is marked attenuation of the increases in corticospinal excitability otherwise obtained in placebo conditions (Korchounov and Ziemann, 2011).

### The serotonergic system—a modulator of neuroplastic adaptation

Batsikadze et al. (2013) administered 20 mg of the selective serotonin reuptake inhibitor (SSRI) citalopram 2 h prior to the commencement of PAS. In the presence of the drug there was a failure to obtain the diminution of MEP amplitude otherwise obtained in the 30 min following PAS10. There was however no consistent impact of citalopram on the usual excitability enhancing effects of a PAS25 protocol.

### The adrenergic system—a modulator of neuroplastic adaptation

The mode of action of methylphenidate is such that it leads to increased extracellular concentrations of both norepinephrine (i.e., noradrenaline) and dopamine. As noted above, it has no apparent influence on the effects of N20+2 PAS (Korchounov and Ziemann, 2011). Prazosin is an alpha-adrenergic antagonist that is specific for the alpha-1 receptors. The prior delivery of 1 mg of the drug eliminates the increases in MEP amplitude otherwise induced by a N20+2 protocol (Korchounov and Ziemann, 2011).

### A reflection on pharmacological studies of PAS-induced effects

Pharmacological studies such as those described above are conceptually powerful in so much as they offer the prospect of causal inference with respect to cellular pathways that are necessary for realizing the effects of non-invasive stimulation protocols such as PAS. In practice there are caveats. These agents—which are typically introduced by oral administration, act at a systems level i.e., not only upon the neural circuits that may be engaged by a particular intervention. In addition, the drugs used most often in human experimentation do not have an exclusive mode of action. It has been highlighted previously (e.g., Paulus et al., 2008) that strong inferences can generally only be drawn in circumstances in which a set of drugs sharing a specific mode of action exhibit consistency in their effect upon the phenomenon that is the focus of interest. Furthermore, effective blinding of participants is often precluded by the side effects of these agents that may include nausea (e.g., Wolters et al., 2003; Monte-Silva et al., 2009; Korchounov and Ziemann, 2011) and sedation (e.g., Korchounov and Ziemann, 2011). There is a paucity of replication studies, and in only a very small number of investigations have dose dependencies been examined. Indeed, ethical considerations necessarily impose limits on the dosages of many drugs that can reasonably be employed with human volunteers.

These matters notwithstanding, is it possible to discern patterns of variation that intimate the cellular mechanisms mediating responses to PAS. With respect to the notional drivers of neuroplastic adaptation, drugs (with the exception of topiramate) that enhance the effects of GABA lead to diminution of the increases in excitability otherwise brought about by N20+2 PAS protocols. Dextromethorphan acts in part as an NMDA receptor antagonist. Its administration blunts the impact of both PAS25 and PAS10 interventions. In relation to drugs that disrupt the action of voltage-gated calcium channels, the effects of N20+2 PAS are diminished by levetiracetam, and those of PAS10 are reduced by nimodipine. Taken at face value, these studies suggest that the effects of both excitatory *and* inhibitory PAS protocols are dependent on both NMDA receptor activation and voltage-dependent Ca^2+^ channels (cf. Muller-Dahlhaus et al., 2010). In addition, they indicate that GABAergic circuits may also play a regulating role in relation to (corticospinal) excitability enhancing forms of PAS. In this regard, there is as yet no information readily available concerning GABAergic mediation of excitability diminishing variants.

Although designated a *modulator* of neuroplastic adaptation, as revealed by the impact of D2/D3 receptor antagonists, the dopaminergic system appears to assume a necessary role in relation to the changes in corticospinal excitability brought about by PAS. It is also notable that the administration of levodopa provides one of the few instances (Kuo et al., 2008) in which a pharmacological agent accentuates the effects of PAS (see also Kuo et al., 2007). Nonetheless, the complex influence of this particular agent and D2 receptor agonists, in particular the presence of non-linear dose-response relationships, precludes a simple interpretation of the part played by dopamine. The role of the cholinergic system is similarly elaborate. At least one cholinesterase inhibitor appears to enhance the effects of both excitatory and inhibitory PAS protocols. In addition, the nAChR receptor agonist nicotine selectively dissipates the inhibitory influence of PAS10, whereas the mAChR receptor antagonist Biperiden has a similar impact on the efficacy of an excitatory N20+2 protocol. With respect to the serotonergic system, at least one agent that increases the extracellular level of the neurotransmitter impedes the inhibitory influence of PAS10. Concerning the adrenergic system, alpha-adrenergic blockade exerts an attenuating influence on the otherwise excitatory effects of N20+2 PAS.

Taken together, these studies paint a picture of multiple cellular mechanisms acting via a complex web of relationships that together mediate the changes in corticospinal excitability induced by both excitatory and inhibitory variants of PAS. The current state of knowledge concerning the cellular foundations of PAS-induced neuroplastic adaptation is sufficiently impoverished that predictions in relation to the outcome of any particular pharmacological perturbation are often usurped by the experimental data. For example, dopamine, norepinephrine, and acetylcholine receptor agonists fail to further augment PAS-induced effects in a context in which there is unlikely to have been saturation of corticospinal excitability (Korchounov and Ziemann, 2011). In light of the conclusion that multiple cellular pathways are almost certainly involved in giving expression to the effects of PAS (e.g., Muller-Dahlhaus et al., 2010; Hamada et al., 2012), we turn our consideration now to mechanisms through which the constituent elements of PAS (i.e., peripheral and cortical) may exert their influence.

## Constituent elements of PAS—sensory stimulation

On the basis of information derived using neuroimaging techniques, the conclusion has been drawn that the form of peripheral afferent stimulation applied in PAS protocols, first engages circuits in the primary somatosensory cortex (S1) within the postcentral gyrus, the second somatosensory area (S2) within the parietal operculum, and the posterior parietal cortex (Korvenoja et al., 1999; Boakye et al., 2000). In relation to mediating the effects of PAS, the temporal characteristics of this engagement are particularly salient. Electrical stimulation of peripheral afferents elicits complex cortical responses that are discernible as SEPs in scalp EEG recordings, and as somatosensory-evoked fields when magnetoencephalography (MEG) is used. There is widespread agreement that the earliest N20 SEP response following electrical stimulation of the median nerve, arises from contralateral (S1) Brodmann area 3b. The balance of evidence now also suggests that the P22 SEP component has its origin in Brodmann area 1 (i.e., S1), rather than for example M1 (Baumgartner et al., 2010). Indeed, a S1 source is in general presumed for short-latency potentials occurring within the first 40 ms following the median nerve stimulus (Allison et al., 1991). Nonetheless, the presence of synchronized neuronal population activity in S2 (registered by MEG) at these latencies, while suggesting an influence of cortical afferents from SI, does not preclude a presence of additional parallel thalamocortical projections to S2 (Karhu and Tesche, 1999). Although there is not yet consensus in relation to the medium latency (>40 ms) components, a distributed pattern of activation that includes not only S1, but also S2 bilaterally, and contralateral posterior parietal cortex is indicated (Hari et al., 1984; Allison et al., 1989a,b, 1992; Forss et al., 1994). These sources continue to be active simultaneously during a period 70–140 ms following the onset of stimulation (Mauguiere et al., 1997). In addition, when trains of afferent stimulation are applied, the offset of the train gives rise to a (P100 and N140) SEP signature distinct from that associated with the individual stimuli (Yamashiro et al., 2008, 2009).

With respect to these temporal features, it is must be emphasized that SEPs (or fields) do not afford unambiguous interpretation. It is well-established that in order to create electrical fields large enough to propagate through the brain, dura, skull, and skin, in the order of 10^7^ of neurons must be active simultaneously. While it is clearly possible to isolate and measure modulations of averaged SEP waveforms generated by the mass action of many neurons, it can be argued that such features as the latency of the peak are arbitrary are no more representative of the temporal dynamics of the latent neural processes than the beginning or end of the deflection (Luck, 2005). It is also typically the case that the voltage fluctuations of the components of a SEP waveform inherently overlap with each other in time and space (see Woodman, 2010, for a review). A deeper problem arises from the corresponding implication that it is not possible on the basis of EEG or MEG measurements to infer temporally discrete propagation of a response to a unitary stimulus (Luck, 2005). These issues have implications not simply in relation to the interpretation of somatosensory-evoked field and potentials, they are pertinent to assumptions that might be made concerning the time course over which peripheral afferent stimulation exerts its effects in the context of PAS.

Ambiguity in relation to the routes via which, and the time course over which, the afferent component of PAS protocols might exert its influence upon the output circuits of primary motor cortex is compounded by the customary use of levels of stimulation above MT. The majority of PAS studies employing mixed nerve targets have used an intensity defined as three times perceptual threshold (e.g., Stefan et al., 2000; Wolters et al., 2003; Sale et al., 2007; Tecchio et al., 2008), which corresponds to a level at which motor potentials are generated (Litvak et al., 2007; Kennedy and Carson, 2008). In the case of trains delivered to the motor point of the target muscle, the stimulation intensity is defined explicitly in relation to the evocation of a visible muscle contraction (Ridding and Taylor, 2001; Castel-Lacanal et al., 2007; Kennedy and Carson, 2008; Carson et al., 2013). Necessarily therefore, in addition to the initial ascending afferent volley induced directly by electrical stimulation of the nerve, all current PAS protocols are likely to encapsulate secondary reafference arising from muscle contractions (Schabrun et al., 2012). The extent of the neural activity induced in M1 by such reafference can be substantially greater than that brought about by the direct sensory consequences of peripheral stimulation (Shitara et al., 2013).

There is in addition a related body of evidence concerning the effects of manipulating the intensity of (electrical) peripheral afferent stimulation. When registered using fMRI, contralateral S1 activity scales with levels of stimulation (at least up to MT) (see also Nelson et al., 2004). The bilateral response obtained for S2 and in posterior parietal cortex does not vary in this manner, although a BOLD response in S2 is registered at lower levels of stimulation than in S1, and is augmented when the participant's attention is directed explicitly to the stimulus (Backes et al., 2000). Similarly, Smith et al. (2003) reported a dose-response relationship for the S1 BOLD response when stimulation was delivered over the quadriceps muscle. Furthermore, the representational overlap of adjacent fingers derived from the BOLD signal in different subdivisions of S1 increases as the intensity of single digit electrical stimulation is increased (Krause et al., 2001).

When median nerve stimulation (0.2 ms pulse) is delivered at 2 Hz, in a range between the sensory threshold (ST) and 1.2 times MT, the amplitude of the components N9, N20, and N20-P25 SEP components increases in proportion to stimulation intensity (cf. Lakhani et al., 2012; Gatica Tossi et al., 2013), an effect that remains evident at 2.5 times MT (Urasaki et al., 1998). While components of the S1 SEP appear to saturate at some point below the pain threshold (PT) (Parain and Delapierre, 1991), MEG recordings suggest that the asymptote of the S2 response occurs at lower stimulation intensities than for the S1 response (Lin et al., 2003). In general the relationship between stimulus intensity and the overall magnitude of the SEP can be characterized as a decelerating power function (Hashimoto et al., 1992). This process, whereby small and desynchronized peripheral volleys are manifested as synchronous cortical potentials has been referred to as CNS amplification (Eisen et al., 1982; Urasaki et al., 1998). Since there is no difference in the extent to which this phenomenon is expressed in the P14 potential that originates from the cervicomedullary junction and the cortical N20/P25 component, it has been concluded that the amplification arises at the cuneate nucleus, and is maintained at the level of S1 (Urasaki et al., 1998).

Studies in cat indicate that stimulation of sensory cortex can induce long-lasting potentiation of synaptic potentials evoked in the motor cortex (Sakamoto et al., 1987). Elevated activity registered by fMRI (Spiegel et al., 1999) and by MEG (Kawamura et al., 1996) is evident in both contralateral S1 and M1 when median nerve stimulation at motor threshold intensity is used. It is notable therefore that when the intensity of peripheral nerve stimulation applied in humans is between 30 and 50% of that required to produce a maximum compound muscle action potential (M-max i.e., well in excess of that used in SAI paradigms), MEPs-evoked subsequently by TMS over M1 are facilitated at ISIs from 25 to 60 ms in APB (following median nerve stimulation at the wrist), and at ISIs from 40 to 65 ms in flexor hallucis brevis (following TN stimulation at the ankle) (Deletis et al., 1992). A similar outcome was noted (Komori et al., 1992) for the thenar muscle at ISIs between 50 and 80 ms when the peripheral shock was set to 10% of M-max. Devanne et al. (2009) reported than even when stimulation intensity is set just above motor threshold, median nerve stimulation (at the wrist) gives rise to marked facilitation of MEPs recorded in APB, FDI, and ECR when ISIs ranging from 40 to 80 ms are employed. When corticospinal excitability is assessed prior to and following the delivery of extended (up to 2 h) sequences of (electrical) peripheral afferent stimuli resembling those used in PAS protocols, intensities close to MT tend to induce facilitation (Kaelin-Lang et al., 2002; Charlton et al., 2003). These findings are consistent with other indications that given a sufficient intensity of afferent stimulation—whether this is achieved by increases in the current/voltage of individual shocks, and/or by a higher frequency of delivery increases in the excitability of corticospinal projections from the primary motor cortex can be induced by this means alone (e.g., Ridding et al., 2000; Khaslavskaia et al., 2002; McKay et al., 2002; Knash et al., 2003; Chipchase et al., 2011; Schabrun et al., 2012, see also Luft et al., 2002).

While the magnitude and duration of the increase in corticospinal excitability induced by PAS25 scales with the number of stimulus pairs (Nitsche et al., 2007), we are not aware of any instances in which the intensity of the afferent stimulation has been manipulated systematically in this context. In so much as the impact of an afferent volley on M1 excitability appears to be proportionately greater for stimulation of nerves in the lower limb than for those in the upper limb—at levels that are ostensibly equivalent when defined in relation to perceptual thresholds (Roy et al., 2007), it may however be possible to derive an indirect indication of the impact of this factor on the effectiveness of PAS protocols. For example, a 30 min period of CP nerve stimulation is sufficient to bring about sustained increases in the excitability of corticospinal projections to TA (Khaslavskaia et al., 2002; Knash et al., 2003), whereas periods of more than 1.5 h are required to induce similar changes in the state of projections to intrinsic hand muscles (e.g., Ridding et al., 2000). It has been noted previously by Roy et al. (2007) that this difference in the potency of the sensory element of PAS may account for the observation that the range of ISIs that is effective for lower limb induction protocols (≈80 ms) is larger than that which is efficacious for muscles in the upper limb (≈35 ms). The more general point to be made is that in addition to the relative timing of its delivery in relation to TMS, the intensity of afferent stimulation may play an instrumental role in determining the magnitude of the effects induced by PAS.

## Constituent elements of PAS—cortical stimulation

There exist a number of authoritative reviews concerning the impact of TMS upon corticospinal output (e.g., Huerta and Volpe, 2009; Siebner et al., 2009; Di Lazzaro and Ziemann, 2013). For the present purposes we draw selectively upon this existing body of knowledge, highlighting those features that may be particularly relevant in relation to PAS. TMS evokes high-frequency repetitive discharge of corticospinal neurons. When it is delivered at intensities above the threshold necessary to evoke a motor response in a peripheral muscle, epidural recordings reveal a series of four or more descending volleys each separated by ≈1.5 ms (see Ziemann and Rothwell, 2000; Di Lazzaro et al., 2012; Di Lazzaro and Ziemann, 2013; for reviews). The first of these is thought to originate from the direct (“D”) activation of corticospinal axons in the subcortical white matter. Those occurring subsequently are believed to require mediation of the cortical gray matter, and to arise from indirect (“I”) trans-synaptic activation of corticospinal neurons.

In PAS induction protocols, it is customary (at least for the upper limb) to employ a relatively focal figure-of-eight stimulating coil, and an angle of application such that the current induced in the brain flows in a posterior to anterior (PA) direction. At sub-threshold stimulation intensities, this configuration yields a single I1 wave that is believed to arise from the action of monosynaptic corticocortical connections projecting onto corticospinal neurons (Di Lazzaro et al., 2008). At the higher stimulation intensities utilized in the administration of PAS (e.g., able to generate a MEP of 1 mV in a hand muscle), further volleys denoted “late I waves” are also generated. It is understood that this repetitive discharge is produced by the activation of complex chains of interneurons that project ultimately onto corticospinal cells (Di Lazzaro et al., 2009a,b). The conclusion that the mechanisms generating these late I-waves are at least partially independent of those giving rise to I1 waves is supported by the observation that the former are suppressed by GABAa receptor inhibitors, whereas I1 waves are not (Di Lazzaro et al., 2008). Of particular note in the present context, the inhibition of the compound MEP produced by the SICI protocol is accompanied by suppression of late I-waves, whereas this is not the case for the I1 wave (Di Lazzaro et al., 1998; Hanajima et al., 1998). A similar differentiation is obtained when LICI protocols employing ISIs of 100 and 150 are employed (Di Lazzaro et al., 2002). Furthermore, SAI of the MEP generated by TMS delivered 1–8 ms after stimulation of the median nerve N20 potential is accompanied by depression of the I2 and I3 waves, whereas the I1 component of the descending volley is relatively unaffected (Tokimura et al., 2000). Taken together, these studies indicate that the M1 networks activated by single pulse TMS at the intensities used customarily in PAS protocols generate I1 waves which contribute to the compound MEP—the standard measure of the efficacy of PAS, in a manner that is relatively impervious to experimental manipulation; and a series of later I-waves that are subject to the modulatory influence of inhibitory GABAa receptor mediated interneuronal networks. Although PAS25 does not alter expressions of SICI or SAI, it increases the amplitude of late I-waves (Di Lazzaro et al., 2009a), whereas PAS10 (which may decrease SICI) appears to decrease their size (Di Lazzaro et al., 2009b). Is it sufficient therefore to restrict consideration of the TMS component of the PAS protocol to its effect on these chains of interneurons with fixed temporal characteristics that produce a periodic bombardment of corticospinal neurons (Amassian et al., 1987), or do its (spatially and temporally) distributed effects also have to be taken into consideration?

There is a large and rapidly expanding literature that concerns the use of imaging techniques such as PET (Fox et al., 1997; Paus et al., 1997), fMRI (Bohning et al., 1997), and EEG registered potentials (ERP) (Ilmoniemi et al., 1997), in conjunction with TMS, to determine patterns of functional brain connectivity. If TMS is applied over a discrete cortical site, the instigated neural activity can be registered as it propagates orthodromically through a network of connected regions (Fox et al., 1997). Augmented by analytic techniques such as structural equation modeling (SEM), which are used to make inferences in relation to causal relationships, these means have be used to determine, for example that there are path connections from primary motor cortex to S2 (bilaterally) (Laird et al., 2008). More generally it has been proposed that the efficacy of protocols such as PAS may depend not only on the characteristics of the stimulated regions, but also upon other elements of the brain network to which they are interconnected (e.g., Cardenas-Morales et al., 2013).

With respect to the “local” effects of TMS, it has variously been adjudged that the spatial extent of the cortical surface that is stimulated extends to 1 cm^2^ (Cowey and Walsh, 2000; Wagner et al., 2004; Thielscher and Wichmann, 2009), although these estimates are increased somewhat when conductivity along the major fiber tracts is taken into account (De Lucia et al., 2007). In relation to temporal extent, when studied in cat, single magnetic stimuli applied to visual cortex give rise to episodes of enhanced and suppressed single-unit activity in the context of a general facilitation that persist for 500 ms (Moliadze et al., 2003).

In relation to the distributed effects of TMS in humans, it is apparent that the delivery of single pulse TMS to M1 produces a complex spreading pattern of activation that can be registered (e.g., by EEG) over 300 ms as it encompasses ipsilateral motor, premotor, and parietal regions (Ilmoniemi et al., 1997; Komssi et al., 2002). It is composed of a sequence of negative deflections peaking at ~7, 18, 44, 100, and 280 ms, alternating with positive peaks at ~13, 30, 60, and 190 ms post-TMS (Ferreri et al., 2011, 2012). In evidently triggering polysynaptic circuits, variable delays will be introduced with the result that the TMS will have distinct effects at different synapses (Huber et al., 2008). It now also accepted that the state of the cortex at the time of the TMS (i.e., when conditioned by peripheral afferent input) both determines the overall neuronal response of the stimulated cortex (Ferreri et al., 2012, 2013), and shapes the responsiveness of distinct subpopulations of cortical neurons (Siebner et al., 2009). Thus, the spatial propagation of TMS invoked coherence, and the functional consequences of this spread of synchronized activity, is contingent upon prior events, and indeed upon those immediately following.

On the basis of observations that rTMS delivered at intensities below motor threshold failed to elicit a discernable BOLD response in M1, whereas suprathreshold intensities consistently do so (Baudewig et al., 2001; Bestmann et al., 2004), Lang et al. (2006) have argued that regional changes in synaptic activity induced by magnetic cortical stimulation are driven in large measure by re-afferent feedback arising from the associated muscle contractions. Applying single pulse TMS (<0.2 Hz) Hanakawa et al. (2009) arrived at a similar conclusion having noted that in the directly stimulated M1, elevated BOLD activity was registered only when intensities above motor threshold were applied. In this context, a bilateral elevation of activity in S2 [plus ventral SMA, caudal cingulate zone (CCZ), and bilateral PMd] was also observed. The BOLD response in ipsilateral S1 was enhanced by both subthreshold and suprathreshold intensities of M1 stimulation (Hanakawa et al., 2009). Recent investigations suggest that at least 10% of the BOLD signal change registered in M1 following suprathreshold TMS is attributable to inputs from muscle afferents (Shitara et al., 2013). As we argued above in relation to the effects of the peripheral stimulation, since standard PAS protocols deliver TMS at intensities above motor threshold, it may be assumed that the associated muscle contraction will give rise to a subsequent reafferent volley that is delayed by tens of milliseconds relative to the initial cortical stimulation and maintained for an extended period thereafter. Necessarily this will lengthen the interval over which the TMS pulse may exert an influence on processes that mediate neuroplastic adaptation.

A handful of studies have been conducted with an explicit focus upon variations in functional connectivity engendered by PAS. Huber et al. (2008) observed that changes in TMS-evoked cortical EEG responses (induced by PAS25 and PAS10 interventions) were expressed for up to 200 ms following the magnetic probe. The largest effects were obtained for the region in which SEPs induced by median nerve stimulation overlapped with TMS-evoked potentials (TEPs). An analysis of movement related cortical potentials (MRCP) obtained prior to and following a N20+2 PAS protocol (15 min duration) suggests that the effects of the intervention also extend to disruption of movement-related effective connectivity between PMd and M1 (Lu et al., 2009). Employing the Ridding and Taylor (2001) protocol that utilizes trains of afferent stimulation, Tsuji and Rothwell (2002) noted increases in the cortical N20/P25 (recorded 2 cm posterior to C3—parietal) and P25/N33 (recorded 5 cm anterior to C3—frontal) components of the SEP for 10 min post-intervention, providing further evidence that PAS-induced changes are both spatially and temporally distributed.

The precise nature of the relationship between the immediate local effects of TMS and the distributed changes in network reactivity that follow remain to be resolved (Shafi et al., 2012). Nonetheless, when applied in the context of a PAS protocol, it is clear that this mode of brain stimulation gives rise to consequential variations in neural activity that are not localized in either space nor time.

## Mediation of interactions between the constituent elements of PAS

In light of the foregoing analyses of the constituent elements of PAS, an assumption that there is discrete temporal convergence of activity generated by the two associated sources of stimulation cannot necessarily be sustained. Consideration might therefore be given to the various routes through which neuronal activity generated by TMS applied to M1, and by peripheral nerve stimulation, may converge and interact.

### Cortico-cortical connections from somatosensory cortex to M1

In spite of initial controversy (Gandevia et al., 1984; Halonen et al., 1988) concerning the relative contribution of muscle afferents and cutaneous fibers to SEPs evoked by electrical stimulation of mixed nerves (e.g., median nerve at the wrist), there is now consensus that the initial (i.e., N20) responses are dominated by cutaneous rather than muscle afferent input (Gandevia and Burke, 1990; Kunesch et al., 1995). On the other hand, EEG potentials associated with movement-generated reafference are largely contingent on input from muscle spindles. The origin of the N20 response to cutaneous inputs is taken to be a deep tangential generator in area 3b (e.g., Desmedt and Ozaki, 1991; McLaughlin and Kelly, 1993). This is consistent with the characteristics of area 3b that have been defined on the basis of comparative studies in primates (Kaas, 1983). It is likely that the source generator for cortical potentials invoked by muscle spindle afference is principally area 3a, although additional contributions from area 2 cannot be excluded (Mima et al., 1996; Mackinnon et al., 2000). This is likewise consonant with the interpretation drawn from comparative studies that that the major driving input to area 3a is from muscle spindle afferents (Kaas, 1983). Thus, the form of peripheral nerve stimulation that is applied in PAS—consisting of an electrical shock (or series) of shocks, will give rise to cutaneous afferent mediated activity in area 3b of primary somatosensory cortex (SI), and also to activity in area 3a and area 2 (Wiesendanger and Miles, 1982) by virtue of contraction induced reafference brought about by the use of stimulation intensities above motor threshold.

Studies in primates (e.g., Jones et al., 1978; Pons and Kaas, 1986; Ghosh et al., 1987; Huerta and Pons, 1990) and in cat (Grant et al., 1975; Zarzecki et al., 1978; Waters et al., 1982; Burton and Kopf, 1984; Yumiya and Ghez, 1984; Porter and Sakamoto, 1988; Avendano et al., 1992) reveal an extensive network of cortico-cortical connections between SI and primary motor cortex (M1) (Burton and Fabri, 1995). Only cells in the superficial layers of M1 (II and III) exhibit short-latency EPSPs—indicative of direct input, in response to microstimulation of area 2 (Kosar et al., 1985; Porter et al., 1990). In contrast, neurons that receive short latency input from area 3a are found in all laminae of the motor cortex, with the exception of layer I (Herman et al., 1985; Huerta and Pons, 1990; Porter et al., 1990). Indeed it has variously been suggested that area 3a should be regarded at the very least as a relay to motor cortex (Jones and Porter, 1980), or even as a part of area 4 (Jones et al., 1978). Regardless of classification, this organization provides a means through which muscle spindle input that is relayed through area 3a can exert a direct influence on pyramidal and multipolar neurons in deep (V and VI) layers of M1 (Porter et al., 1990). Since the former are suspected to have a facilitatory, and the latter an inhibitory influence on corticofugal cells, this may account, in part, for the alternating pattern of MEP facilitation and inhibition that is observed in response to peripheral nerve stimulation at ISIs shorter than 80 ms (Sailer et al., 2002). Relays involving corticocortical input from area 2 to pyramidal cells in layers II/III of M1 (Kaneko et al., 1994a,b) and then to layer V/VI pyramidal neurons (Kaneko et al., 2000) may also play a role in this regard.

In marked contrast, while there are reciprocal connections between area 3b and area 1 in particular, and further projections to area 2 (which are seemingly not reciprocated), projections from area 3b to M1 are sparse (Darian-Smith et al., 1993; Burton and Fabri, 1995), if present at all (Jones et al., 1978). This being the case, it worth reflecting upon the use of the N20 response latency—which is presumed to have a generator in area 3b, as a reference in determining ISIs in PAS protocols, since this region has few if any direct projections to M1, and is not engaged to a significant degree by peripheral input from muscle spindle afferents. When the interval between mixed nerve stimulation and TMS delivered over *S1* is adjusted with a view to ensuring that the cortical stimulus occurs around the time that reafference arising from the muscle contraction is maximal (N20 + 100 ms), changes in tactile sensitivity otherwise observed when the cortical and peripheral stimulation both occur within a 20 ms window are no longer obtained (Litvak et al., 2007). Since the former condition also gives rise to a medial shift in the topography of the multi-channel SEP, these authors concluded that the reafference driven effects observed in the N20 + 100 associative protocol were expressed via selective enhancement of a source in area 3a, whereas when shorter ISIs were employed, a source in area 3b was also implicated (Litvak et al., 2007). It might also be remarked that PAS protocols employing digital nerve stimulation—which may excite mechanoreceptor fibers rather than muscle spindle afferents (and TMS to M1), tends to produce smaller effects than mixed nerve stimulation (e.g., Stefan et al., 2000; Kujirai et al., 2006). Thus, to the extent that muscle spindle afferents represent the most efficacious source of peripheral input in PAS protocols, it may surmised that area 3a—which has projections onto neurons in most layers of M1—including laminae II/III which are believed to represent the origin of late I-waves [and receives substantial inputs from S1 (Huffman and Krubitzer, 2001)], may play a critical role in mediating the changes in corticospinal output that are induced.

Although a direct activation of the motor cortex via sensory afferents from the periphery (Padel and Relova, 1991) cannot be dismissed, studies in monkey demonstrate that the ventral posterior complex of the thalamus, the major sensory thalamic relay, only has minor direct projections to the motor cortex (Darian-Smith and Darian-Smith, 1993; Huffman and Krubitzer, 2001) and thus a structural correlate for direct motor cortex activation after peripheral sensory stimulation has not yet been found. In addition, as highlighted above, while it has been proposed that the P22 SEP component may originate from the precentral motor area, the balance of evidence now indicates that the source is in area 1 (i.e., S1) (Baumgartner et al., 2010). It is also worth noting in this context that while S1 areas 1, 2 and are represented across the ventrobasal complex of the thalamus, area 3a has connectional relationships similar to those for area 4 (Jones et al., 1979), further emphasizing the likelihood that muscle afferent input relayed via area 3a will have a more direct influence on the state of the primary motor cortex, than cutaneous input relayed via other regions of S1.

### Cerebello-thalamo-cortical and thalmo-cortical connections

The point has been made previously that functional neuroplastic adaptation is likely to encompass changes in activity distributed across “non-primary” elements of the sensorimotor network, including the supplementary motor area and lateral premotor cortex, cingulum, insula, posterior parietal cortex, cerebellum, deep gray nuclei and thalamus (Duffau, 2006). As a case in point, as the VL nucleus is the primary relay station in the cerebello-thalamo-cortical pathway (Asanuma and Hunsperger, 1975), it has been proposed that, through receipt of convergent inputs from both the sensorimotor cortex and the spinal cord, the interpositus nucleus of the cerebellum exerts a modulating influence upon motor network responses to sensory stimulation via thalamic projections to premotor and motor cortices (Luft et al., 2005). In this vein, hemicerebellectomy blocks the modulation of cortical motor output associated with repetitive electrical stimulation of the sciatic nerve in the rat (Ben Taib et al., 2005). The state of the motor cortex itself—acting via the intermediate cerebellum, may further serve to tune the gain of polysynaptic responses to peripheral stimulation (Manto et al., 2006). Hamada et al. (2012) demonstrated recently that when either anodal or cathodal transcranial direct current stimulation (tDCS) was applied to the cerebellum during a PAS25 protocol, the usually obtained excitability enhancing effect of this intervention was not exhibited. In contrast, anodal tDCS failed to modulate the impact of a PAS21.5 protocol. Popa et al. (2013) reported that 600 prior rTMS stimuli applied over the posterior cerebellar cortex in either a continuous (cTBS) or an intermittent (iTBS) theta burst pattern, has opposing effects on the efficacy of PAS protocol. The iTBS pattern attenuated the increases in corticospinal excitability brought about by PAS, whereas the cTBS pattern enhanced and prolonged increases in MEP amplitude.

Area 3a of the primary somatosensory cortex receives projections from nuclei of the thalamus classically associated with the motor system, including indirect input from the cerebellum and basal ganglia via the ventral lateral (VL) nucleus (Huffman and Krubitzer, 2001). Thalamic processing of somatosensory input appears however to extend beyond the relaying of primary afferent signals to the cortex. For example, at levels of median nerve stimulation above PT, thalamic SEPs can be elicited for longer than 75 ms after the peripheral shock, with this duration extending to 150 ms when the intensity is set to MT (Klostermann et al., 2009).

It has been noted previously that since afferent input is relayed via the cerebellum and VL to area 4, and inputs from the globus pallidus reach M1 after relaying in VL, the motor cortex is capable of influencing both its own thalamic afferents and those directed to the primary somatosensory cortex (Canedo, 1997). Indeed, low frequency electrical stimulation of the sensorimotor cortex evokes short and long-latency excitatory and inhibitory postsynaptic potentials in VL neurons. In this regard, it has been proposed that the thalamocortical cells operate in two different modes: an oscillatory mode and a tonic (transfer) mode. The particular significance of this characteristic in the present context is the facility for corticothalamic fibers to induce thalamic oscillating activity that renders the thalamic neurons unresponsive to synaptic input—functionally deafferenting the cerebral cortex (Canedo, 1997). It remains to be determined whether TMS applied to M1 is capable of blocking afferent transmission via the thalamus in this fashion. If so, it may cast a different light on the means through which such protocols as PAS10—whereby TMS precedes the cortical corollary of the peripheral stimulus, serves through repetition to decrease the excitability of projections from M1 (see also Schabrun et al., 2012). While it is known that TMS can suppress the perception of subsequent peripheral afferent stimuli (McKay et al., 2003; Yoo et al., 2008), it is not yet possible to exclude the possibility that this phenomenon is due to sensory masking rather than to gating of the ascending volley, for example at the level of the thalamus.

It has been conjectured that a disruption of basal ganglia-thalamocortical loops arising from striatal dopamine depletion may account for the reduced response to PAS25 that is exhibited by patients with Parkinson's disease (Ueki et al., 2006), and in the course of normal ageing (Fathi et al., 2010). As yet however, this proposition has not been studied in detail. It can nonetheless be concluded that there are a number of neural circuits extending beyond the S1-M1 axis, encompassing cerebello-thalamo-cortical and thalmo-cortical pathways that have the potential to mediate changes in M1 excitability brought about by PAS.

## Mechanisms of neuroplastic adaptation engaged by PAS

In reflecting upon the characteristics of the data obtained in their seminal study of PAS, Stefan et al. (2000) emphasized that changes in the excitability of human motor cortex brought about by such exogenous stimulation are likely to proceed via a number of different routes. For example, these authors highlighted the possibility that variations in membrane excitability, such as those demonstrated in experiments concerning conditional learning (Woody and Engel, 1972; Aou et al., 1992), may play an instrumental role. Other commentators have also been careful to acknowledge that a range of cellular mechanisms may be engaged (e.g., Muller-Dahlhaus et al., 2010; Nitsche et al., 2012). Why then is it the case that interpretations of PAS framed in terms of STDP are so pervasive? In the present section we will consider whether the empirical evidence presented above, and more general considerations in relation to the complexity of the *in vivo* human motor system, support this narrow emphasis.

### Spike timing dependent plasticity (STDP)

LTP and LTD can be induced by a wide variety of experimental protocols. It has been suggested (e.g., Wolters et al., 2005) that STDP occupies a unique position in so much as the polarity of the induced change in synaptic efficacy is determined by the sequence of pre- and postsynaptic neuronal activity (for reviews see Dan and Poo, 2004; Markram et al., 2011). In the classical model of STDP (e.g., Song et al., 2000), strengthening (potentiation) arises if the presynaptic neuron fires no more than 50 ms in advance of the postsynaptic neuron (Feldman, 2000), whereas weakening (depression) occurs if postsynaptic spikes precede presynaptic action potentials—(or transpire without activity in the presynaptic neuron) (Levy and Steward, 1983; Bi and Poo, 1998; Cooke and Bliss, 2006). In addition, there is a sharp transition from strengthening (LTP) to weakening (LTD) at time differences in the region (within 5 ms) of zero (Feldman, 2012).

At most glutamatergic synapses in the CNS the NMDA receptor (i.e., post-synaptic) performs the function of coincidence detection. The binding to AMPA receptors of glutamate released by presynaptic activation, and the resulting postsynaptic depolarization which leads to removal of the Mg2+ block, together permit the influx of Ca2+ though the NMDA receptors (Mayer et al., 1984; Nowak et al., 1984). The magnitude and time course of the calcium flux (and Mg2+ kinetics) determines whether LTP or LTD is induced (e.g., Verhoog et al., 2013). Transient, high calcium-fluxes invoke LTP, whereas sustained moderate calcium fluxes generate LTD, and low calcium fluxes do not induce adaptation (Lisman, 1989; Yang et al., 1999).

In itself, this mechanism is not strictly timing-dependent (and thus Hebbian) in nature, as the level of activity at *individual* synapses and the firing of the postsynaptic neuron need not necessarily be correlated (Thickbroom, 2007). Back-propagating action potentials (BAP)—which pass antidromically into the soma and then to the dendritic tree following the initiation of an action potential, appear however to provide the retrograde signal through which a contingent association could be instantiated. In the context of LTP, the arrival of presynaptic input milliseconds before the BAP reaches the dendrite can facilitate removal of the Mg2+ block on NMDA receptors and thus promote Ca2+ influx, although other types of interactions between the EPSP and the BAP cannot be excluded (Caporale and Dan, 2008). Conjectures in relation to the mediating role for the BAP in LTD are based on the assumption that it produces an afterdepolarization, such that the generation of an EPSP leads to only a moderate Ca2+influx through NMDA receptors. Although, as with the induction of LTP, a number of alternative models have also been proposed (Caporale and Dan, 2008). With respect to both LTP and LTD, the primary mechanisms of STDP are putatively postsynaptic, and instantiated via addition or removal of AMPA receptors (AMPARs) and changes in single-channel conductance (Malinow and Malenka, 2002).

### STDP in context

Evidently, there exist forms of neuroplastic adaptation that do not depend on BAPs. In addition, it now widely acknowledged that the relative timing of postsynaptic spikes and presynaptic action potentials is only one of several factors, including firing rate and dendritic depolarization that operate in relation to STDP (Feldman, 2012). If facilitatory and inhibitory forms of PAS differ only in respect of the polarity of STDP (cf. Wolters et al., 2005), one might anticipate that their effects would be expressed via the same electrophysiological measures, and that they would be responsive to the same pharmacological manipulations. As highlighted in the preceding sections however, this is not generally the case. At least two possibilities are thus admitted. In the first instance it is possible that the induction of changes in corticospinal excitability by PAS requires the engagement of cellular mechanisms other than, or in addition to, those associated with STDP. An alternative possibility is that factors that influence the expression of STDP exert differential effects depending on the protocol that is applied (and the neural circuits that are targeted). It is for example, well-established the range of effective timing intervals varies across different modes of stimulation and cell types, as well as across species (Bi and Poo, 2001; Caporale and Dan, 2008).

With respect to interceding factors, the backpropagation of action potentials from the site of initiation on the axon to the dendrites provides a critical element of the associative signal for the induction of STDP (Magee and Johnston, 1997). Yet the full expression of STDP—as opposed to LTD only, requires either the enhancement of BAP propagation, for example by inactivation of A-type potassium channels, and/or additional sources of depolarization, which may include recruitment of dendritic sodium channels (Sjostrom et al., 2001; Sjostrom and Hausser, 2006). In addition, the dendritic tree itself is not static. Rather, it is subject to modification by synaptic activity and by neuromodulators (e.g., Sjostrom et al., 2008). The latter, including norepinephrine and acetylcholine, exert an influence on the BAP by altering the activation and deactivation of various active conductances (Caporale and Dan, 2008). Of perhaps greater significance in the context of PAS is the recognition that while LTP occurs at excitatory glutamatergic synapses, inhibitory projections mediated by GABA also play a significant role in its induction in the hippocampus (Davies et al., 1991; Chapman et al., 1998), and that GABAergic influences must be attenuated for LTP to be induced in motor cortex slices *in vitro* (Hess and Donoghue, 1996). It has been shown recently that the interceding role of GABA extends to STDP. Specifically, the polarity of STDP induced experimentally at corticostriatal synapses in rodents can be reversed by blockade of GABAA receptors (Paille et al., 2013).

Aside from consideration of factors that influence its expression *in vitro*, it is likely that neuroplastic adaptation in general (Daoudal and Debanne, 2003; Zhang and Linden, 2003), and synaptic plasticity in particular (Schulz, 2010), is governed by processes that are much more complex than those traditionally ascribed to STDP. Indeed, the argument has been made persuasively that, *in vivo*, backpropagating action potentials are neither necessary nor sufficient for synaptic plasticity (Lisman and Spruston, 2005). More pointedly these authors have argued that textbook accounts of STDP are sufficiently impoverished as a general model of synaptic plasticity in naturally active neural circuits that they constitute “a dangerous oversimplification” (Lisman and Spruston, 2010). With specific regard to the topic at hand, Thickbroom (2007) has noted that, given the likely temporal dispersion of the nominally coincident inputs contrived in PAS protocols, the effects that emerge are more probably a consequence of increased network activity generated by convergent inputs (i.e., an activity dependent mechanism), rather than STDP. Indeed, there are contemporary models which predict that potentiation will occur during periods of high pre- and post-synaptic activity in a manner that is independent of the temporal order of spikes (Pfister and Gerstner, 2006). Thus, the dependence on timing expressed empirically may reflect the means through which network activity is increased, rather than a signature of STDP.

In animals that are awake, neurons throughout the cerebral cortex have high spontaneous firing rates, and at any given moment multiple synaptic inputs to single neurons are active simultaneously. As a consequence of this high conductance state, the integrative properties of cortical neurons *in vivo* are profoundly different from neurons maintained *in vitro* (Destexhe et al., 2003). The natural system is inherently stochastic such the relationship between input and outputs is probabilistic—defined by the response characteristics of a population of neurons that share common input (Shadlen and Newsome, 1998). On the other hand, many neurons, including pyramidal neurons, have extensive dendritic arborizations and receive and process synaptic input from widespread sources. In the prototypical experiments used to define the canonical characteristics of STDP, unitary EPSPs were evoked by stimulation of a single presynaptic cell, and a single BAP was induced by the brief injection of current into the soma of the postsynaptic cell. Recent work conducted under conditions of spontaneous activity *in vivo* suggest that, with respect to layer V pyramidal neurons in M1, synaptic input and dendritic activity are spread uniformly throughout all branches of the dendritic tree, rather than it being the case that NMDA spikes are localized to a single branch (i.e., functioning as the putative unit of plasticity) (Hill et al., 2013). While to the best of our knowledge there have not yet been corresponding studies focusing on the layer II/III pyramidal neurons in M1 that are thought to mediate the effects of PAS, in light of the foregoing considerations it would seem unlikely if the effects of this form of non-invasive brain stimulation were to be “synapse specific,” at least in the sense in which this term is understood in relation to STDP.

While in the reduced preparations that have typically been used to deduce the characteristics of STDP there is only one connection between the pre- and post-synaptic neurons, at the more macroscopic scale relevant to electrophysiological manipulations and recordings in humans, there are multiple pathways via which sensory corollaries of peripheral stimulation may reach and influence the cortex (Hamada et al., 2012). Similarly, the complex temporal structure of natural neural activity is not reflected in the intermittent pairing of stimuli used to investigate STDP in reduced preparations (Jackson, 2012). Thus, there appears to be little by way of an a priori basis for the assumption that STDP plays a prominent role in mediating the effects of PAS. Furthermore, as Lisman and Spruston (2010) have cautioned in specific relation to this form of neural plasticity, “a field must not go beyond the data” (p. 3).

### The exceptions that may disprove the rule

It has been highlighted previously (Hamada et al., 2012) that there is a general assumption that only the initial element of the input to S1 arising from peripheral afferent stimulation (as reflected in the latency of the N20 component of the SEP) contributes to the changes in corticospinal excitability brought about by PAS. Yet the cardinal phenomenological features also emerge when the stimulation that is associated with TMS cannot be rendered in terms of a discrete series of time locked events (i.e., of fixed latencies).

Thabit et al. (2010) paired TMS (delivered over M1) with each of a series of contralateral thumb abduction movements, initiated in the context of an over-learned visually cued reaction time task (240 pairs at 0.2 Hz over 20 min). When the magnetic stimulus *preceded* the mean individualized RT by 50 ms, the intervention gave rise to increases in the excitability of corticospinal projections to the target muscle that were sustained for up to 15 min. There was a corresponding increase in the duration of the CSP over this period, however no changes in SICI were observed. With respect to these additional features too therefore, the pattern of outcomes resembled that obtained using conventional facilitatory PAS protocols. A decrease in corticospinal excitability was obtained when TMS was applied 100 ms after the mean RT, however no changes were observed when it was followed by 50 or 150 ms, or when the TMS preceded the mean RT by 100 ms. Intracortical recordings in monkey indicate that that pyramidal cell firing increases 150 ms prior to movement initiation (Evarts, 1966, 1968). The time at which TMS was delivered in order to induce sustained facilitation (50 ms prior) therefore falls well within this interval. Although neighboring neurons in M1 with similar output projections may themselves exhibit synchronous firing (Jackson et al., 2003), since the protocol employed by Thabit et al. (2010) provided no control over the temporal relationship between these physiological events and the excitation brought about by the delivery of TMS over M1, an explanation of the associative effects framed in terms of activity dependent mechanism is more parsimonious than one that makes appeal to the rules of STDP.

The requirement for an alternative model is also suggested by a recent study in which the other element of PAS—peripheral afferent stimulation was paired with motor imagery. Mrachacz-Kersting et al. (2012) delivered electrical stimulation (intensity = MT) to the CPN while the participants imagined the kinaesthetics of a ramp and hold dorsiflexion movement, which was cued visually every 10–12 s for a total of 50 pairings. In separate conditions, the timing of the afferent stimulation was delivered 101 ± 110 ms prior to the start of the imagined movement, 134 ± 115 ms after, and 368 ± 196 ms after the start of the imagined movement. Increases in the excitability of corticospinal projections to TA were reported when the peripheral stimulation ~135 ms following the start of the imagined task, but not in the other conditions (see also Niazi et al., 2012). While these exemplars highlight the importance of the timing relationship between the voluntary engagement of motor output networks and TMS delivery and on the one hand, and that between the unfolding of an imagined movement and the administration of peripheral stimulation on the other, in neither instance can the conclusion be drawn that the constituent events are sufficiently discrete (i.e., in terms of timing) to satisfy the requirements of STDP. There are alternative models however that can account for the cumulative impact of temporally proximate events separated by intervals such as those described above (e.g., Ostojic and Fusi, 2013). It remains the case however, that since the prevailing explanatory accounts of the effects of PAS in humans have been framed in terms of STDP, there are as yet few empirical studies that scrutinize the role of other mechanisms in this context.

### What are the features for which any model should account?

If accounts based on STDP, at least as it has been characterized *in vitro*, fail to provide a complete explanation of the effects of PAS in humans, what are the key features that must be encapsulated by alternative models? It is evident for example that the specific intervals (e.g., Schabrun et al., 2013) and the range of intervals (Ridding and Taylor, 2001) that are effective in inducing facilitation or inhibition, vary depending on the corticomotor pathway that is engaged (Roy et al., 2007), and the mode of peripheral stimulation that is employed (e.g., Suppa et al., 2013). Ideally therefore a comprehensive model will encompass this diversity.

It is also apparent that restriction of the effects of PAS to muscles innervated by the peripheral nerve that receives stimulation is the exception rather than the rule. An explanation should therefore be sought for a somatotopic gradient that is relative rather than absolute (e.g., Quartarone et al., 2003). The evidence that has been derived from a limited number of direct investigations suggests that the initial I-wave generated by TMS is not affected by PAS, whereas later I-waves are accentuated by facilitatory, and attenuated by inhibitory variants of the intervention (Di Lazzaro et al., 2009a,b). There is however divergence with respect to other neural circuits through which the effects of PAS are manifested. Excitability enhancing PAS protocols (e.g., PAS25) do not alter expressions of SICI, ICF, or SAI, whereas inhibitory protocols (e.g., PAS10) may decrease SICI. Furthermore, while facilitatory variants decrease LICI and LAI, and elongate the CSP, inhibitory protocols do not appear to influence the CSP. Thus, switches in the polarity of the net change in corticospinal excitability brought about by different variants of PAS are not accompanied by corresponding alterations in the reactivity of neural circuits that act ultimately upon the state of corticospinal neurons. Yet, since the data concerning some aspects of the preceding synopsis are sparse, a number of the characterizations remain incomplete.

With respect to the action of pharmacological agents upon the effects of PAS, NMDA receptor antagonists blunt the impact of both facilitatory (Stefan et al., 2002; Suppa et al., 2013) and inhibitory (Wolters et al., 2003) forms of PAS. It should be noted in this context that a dependence upon NMDA receptors is not an exclusive property of STDP, nor is it restricted to post-synaptic mechanisms. Indeed, NMDA receptor activation has the capacity to enhance inhibitory GABAergic transmission via presynaptic mechanisms mediated by retrograde nitric oxide (NO) signaling (Xue et al., 2011, see also Rodríguez-Moreno et al., 2010; Duguid, 2013). More generally in the context of PAS, drugs that enhance the effects of GABA tend to diminish the increases in corticospinal excitability otherwise brought about by facilitatory protocols. It is well-established that GABA assumes multiple inter-related roles in regulating the excitability of brain networks (Olsen and Sieghart, 2009), encompassing both synaptic activity and extra-synaptic tone (Semyanov et al., 2004). As such, the impact (i.e., on any form of non-invasive brain stimulation) of a drug that interferes with GABA function is subject to a number of possible interpretations. Likewise, the dependence of both excitatory and inhibitory PAS protocols on voltage-dependent Ca^2+^ channels is consistent with both STDP and activity-dependent (Lisman, 1989) models of synaptic plasticity.

It is striking that the dopaminergic system appears to assume a necessary role in relation to the changes in corticospinal excitability brought about by PAS. The influence of dopamine is consistent with a number of different models of neuroplastic adaptation, including those that emphasize stimulus-response contingencies (e.g., Samson et al., 2010), although a specific role of dopamine signaling in relation to STDP has also been mooted (Izhikevich, 2007). Nonetheless, it is apparent that the action of dopamine (and indeed that of other classes of neuromodulator) must necessarily be an element of any comprehensive model of PAS.

## Conclusions

A large body of empirical evidence has accumulated in the period since the cardinal features of PAS in humans were first disclosed, and as variations upon the original protocols have proliferated, deviations from these features have become more numerous. While presenting challenges to assimilation, in seeking to determine the mechanisms that mediate the effects of PAS these variations are a blessing in disguise. Specifically they serve to enforce recognition that the cellular pathways that contribute to the LTP- and LTD-type responses to PAS may differ depending on the precise nature of the induction protocol that is used. For example, in circumstances in which trains of afferent stimulation are applied, it seems likely that classical STDP-type mechanisms will play a diminished role, at least relative to those contexts in which the timing relationship between the peripheral and cortical stimulation can be precisely circumscribed. Furthermore, it need not be assumed that—even within the context of a protocol in which a single parameter such as ISI is manipulated, excitability enhancing and excitability diminishing variants represent a change in the polarity of a specific cellular process. They are not necessarily two sides of the same coin. Indeed, even in relation to a single polarity of effect, relatively minor variations of ISI—in the order of a few milliseconds may alter profoundly the pathways that are instrumentally engaged (e.g. Hamada et al., 2012). The challenge now lies in moving beyond accounts predicated only on the rules of STDP, to encompass the additional physiological mechanisms of action that promote neuroplastic adaptation in natural systems, and appreciate the context-sensitive features of their contributions.

### Conflict of interest statement

The authors declare that the research was conducted in the absence of any commercial or financial relationships that could be construed as a potential conflict of interest.
